# Serum protein pattern associated with organ damage and lupus nephritis in systemic lupus erythematosus revealed by PEA immunoassay

**DOI:** 10.1186/s12014-017-9167-8

**Published:** 2017-10-03

**Authors:** Anna Petrackova, Andrea Smrzova, Petr Gajdos, Marketa Schubertova, Petra Schneiderova, Pavel Kromer, Vaclav Snasel, Martina Skacelova, Frantisek Mrazek, Josef Zadrazil, Pavel Horak, Eva Kriegova

**Affiliations:** 1 Department of Immunology, Faculty of Medicine and Dentistry, Palacky University, Hnevotinska 3, 775 15 Olomouc, Czech Republic; 2 Department of Internal Medicine III - Nephrology, Rheumatology and Endocrinology, Faculty of Medicine and Dentistry, University Hospital, Palacky University, Olomouc, Czech Republic; 3 Department of Computer Science, Faculty of Electrical Engineering and Computer Science, Technical University of Ostrava, Ostrava, Czech Republic

**Keywords:** Serum pattern, Systemic lupus erythematosus, Proximity extension immunoassay, Organ damage, Lupus nephritis

## Abstract

**Background:**

Systemic lupus erythematosus (SLE) is a remarkably heterogeneous autoimmune disease. Despite tremendous efforts, our knowledge of serum protein patterns in severe SLE phenotypes is still limited. We investigated the serum protein pattern of SLE, with special emphasis on irreversible organ damage and active lupus nephritis (LN) as assessed by renal Systemic Lupus Erythematosus Disease Activity Index.

**Methods:**

We used proximity extension immunoassay (PEA, Proseek Multiplex, Olink) to assess the serum levels of ninety-two inflammation-related proteins in Czech patients with SLE (n = 75) and age-matched healthy control subjects (n = 23). Subgroup analysis was carried out on the basis of organ damage (with/without, 42/33) and biopsy-proven LN (with/without, 27/48; active LN, n = 13; inactive LN, n = 14).

**Results:**

Of thirty deregulated proteins between SLE and the healthy controls (*P*_*corr*_ < 0.05), the top upregulated proteins in SLE were sirtuin 2, interleukin 18 (IL18), and caspase 8 (*P*_*corr*_ < 0.0006). Of these, sirtuin 2 and caspase 8 had not yet been reported with SLE. Elevated levels of IL8, CCL2/MCP1, CCL11, and MMP10 (*P*_*corr*_ < 0.05) were detected in patients with organ damage for which the serum levels of CCL11 and MMP10 were particularly informative in organ damage prediction. Comparing patients based on LN, elevated levels of CSF1, sIL15RA, sCD40, sCX3CL1, caspase 8, sIL18R1, bNGF, and GDNF (*P*_*corr*_ < 0.05) were detected in active LN. Except GDNF, all LN-associated markers showed usefulness in prediction of active renal disease.

**Conclusions:**

This highly sensitive PEA analysis identified the serum pattern of SLE, organ damage, and active LN, with many novel candidate proteins detected. Their exact role and suitability as biomarkers in SLE deserve further investigation.

**Electronic supplementary material:**

The online version of this article (doi:10.1186/s12014-017-9167-8) contains supplementary material, which is available to authorized users.

## Background

Systemic lupus erythematosus (SLE) is a serious, complex, multi-system autoimmune rheumatic disease with significant variability in the phenotypes and severity of the disease. The greatest challenges continue to be the prevention and management of irreversible organ damage and active lupus nephritis (LN), one of the most feared phenotypes in SLE.

Organ damage is a primary outcome in SLE, which is accrued not only during the disease course, but also by therapy itself [1]. Early damage is more likely to be linked to active inflammation, while late irreversible damage is often attributable to the side effects of drugs and especially to chronic and cumulative corticosteroid exposure [2]. The Systemic Lupus International Collaborating Clinics/American College of Rheumatology SLICC/ACR Damage Index (SDI), divided into 38 items grouped in 12 organ systems, is a valid measure of irreversible organ damage in SLE [1]. Despite improvement in the survival of SLE patients in recent decades, significantly higher morbidity and mortality are reported in patients developing irreversible organ damage [1]. The patterns of organ damage vary among populations [3–5], but the musculoskeletal, cardiovascular, and renal systems are those most frequently affected [6]. Nowadays, prevention of irreversible damage is a major goal in the management of SLE patients and identification of the key molecules involved in the pathogenesis of organ damage is needed.

Lupus nephritis is a major manifestation associated with higher morbidity and mortality of SLE patients [7]. It has a considerable influence on treatment decisions, as well as long-term outcomes. The effective treatment of LN requires a correct diagnosis, timely intervention, and early treatment of any disease relapse. Renal biopsy is still the gold standard for diagnosis and deciding on therapy in LN but its invasive nature prevents it from being used repetitively in many cases [8]. Traditional clinical parameters such as proteinuria, glomerular filtration rate, urine sediments, anti-dsDNA antibodies, and complement levels are not sensitive or specific enough to detect activity and early relapse of LN [9, 10]. Novel serum and urinary biomarkers such as cytokines and chemokines CCL2 [11], CCL3, CCL5 [12], IL17 [11], BLyS, APRIL [13], growth factor TGFβ [11] and others (TWEAK [14], IGFBP2 [15], OPG [16]) have recently been nominated for diagnosis and monitoring of LN. Although intensively investigated [17, 18], only a few biomarkers have been assessed for prediction of renal activity or prognosis. Identification of novel and reliable biomarkers or their combinations for LN reflecting also disease activity is, therefore, highly desirable.

In this study we aimed to assess the serum protein pattern of SLE using a highly sensitive multiplex proximity extension immunoassay (PEA) on 92 inflammation-related proteins. Special emphasis was given to serum patterns associated with irreversible organ damage and LN reflecting the renal disease activity and their usefulness in the prediction of these severe phenotypes.

## Methods

### Study population and materials

Serum samples were obtained from 75 Czech SLE patients; all enrolled patients fulfilled the ACR classification criteria [19]. The samples were aliquoted and stored at − 80 °C until further use. Organ damage was assessed by means of the SDI damage index (Systemic Lupus International Collaborating Clinics/American College of Rheumatology Damage Index) [1] and disease activity was evaluated by means of SLEDAI (Systemic Lupus Erythematosus Disease Activity Index) [20]. Subgroups were formed on the basis of (1) the SDI (SDI = 0, n = 33; SDI = 1, n = 17; SDI ≥ 2, n = 25), (2) the biopsy-proven presence of LN (no LN, n = 48; LN, n = 27), and (3) the renal SLEDAI within LN subgroup, where renal SLEDAI score of ≥ 4 was taken as an indicator of active LN (inactive LN, n = 14; active LN, n = 13). The renal SLEDAI consists of the four renal parameters: hematuria, pyuria, proteinuria, and urinary casts [20]. The mean of LN duration in active LN patients was 7 years (range 0–19 years) and in inactive LN patients 8 years (range 1–18 years). The demographic and clinical features are described in Table 1. The age-matched control group of healthy subjects comprised 23 medical staff members (mean age 40, range 26–73, female/male 15/8), who gave statements about their health status and excluded any medication used for SLE treatment (corticosteroids, antimalarials, immunosuppressant drugs). The patients and control subjects provided written informed consent about the usage of peripheral blood for the purpose of this study, which was approved by the ethics committee of the University Hospital and Palacky University Olomouc.

**Table 1:** Demographic and clinical characteristics of enrolled SLE patients and subgroups based on the presence of organ damage and LN

Demographic and clinical features	SLE (*n* = 75)	Organ damage (*n* = 42)	No organ damage (*n* = 33)	LN (*n* = 27)	No LN (*n* = 48)
Female/Male	66/9	34/8	32/1	22/5	44/4
Age (years) mean (min–max)	40 (19–74)	44 (20–67)	35 (19–74)	35 (19–57)	46 (25–64)
Age at the onset of the disease (years) mean (min–max)	27 (11–58)	31 (11–58)	26 (12–56)	24 (12–55)	33 (11–58)
Duration of the disease (years) mean (min–max)	11 (1–38)	13 (1–38)	10 (1–31)	11 (1–20)	13 (1–38)
Organ damage (SDI ≥ 2/SDI = 1/SDI = 0)*	25/17/33	25/17/0	0/0/33	10/5/12	15/12/21
Organ damage: SDI mean (min–max)	1.2 (0–8)	2.2 (1–8)	0 (0–0)	1.1 (0–5)	1.3 (0–8)
Lupus nephritis, biopsy proven (Y/N)	27/48	15/27	12/21	27/0	0/48
Neurological involvement (Y/N)^@^	22/53	15/27	7/26	9/18	13/35
Hematological involvement (Y/N)^#^	19/56	15/27	4/29	5/22	14/34
Cardiovascular involvement (Y/N)^§^	12/63	11/31	1/32	4/23	8/40
Skin and musculoskeletal involvement (Y/N)^†^	56/19	28/14	28/5	6/21	35/13
Antiphospholipid syndrome (Y/N)^$^	23/52	15/27	8/25	6/21	17/31
Renal disorder (Y/N)^#^	35/40	19/23	12/21	27/0	9/39
Disease activity: SLEDAI mean (min–max)	7 (0–43)	8.8 (0–43)	4.7 (0–26)	10.3 (0–43)	5.2 (0–20)
Active/inactive renal disease^&^	17/58	12/30	5/28	13/14	4/44
Mean of cumulative dose of glucocorticoids (g) (min–max)	22.8 (0–79.2)	30.6 (2.6–79.2)	12.8 (0–54.0)	27.2 (2.4–68.4)	20.3 (0–79.2)

*SLICC/ACR Damage Index (SDI) was used as a measure of irreversible organ damage in SLE [1]

^@^Defined by the ACR nomenclature [21]

^#^Defined by the ACR classification criteria [19]

^§^Defined as documented pericarditis or myocarditis with compromised left ventrical function or valvular disease

^†^Skin involvement defined by Gillian´s criteria [22] and arthritis by ACR definition [19]

^$^Defined by preliminary classification criteria for antiphospholipid syndrome [23]

^&^Renal SLEDAI score of ≥ 4 was taken as an indicator of active LN [24, 25]

### Proximity extension immunoassay (PEA)

The serum levels of 92 inflammation-related proteins were simultaneously measured by a PEA using the Proseek Multiplex Inflammation kit I (Olink Bioscience, Sweden) according to the manufacturer’s recommendation. Briefly, each analyte is recognized by a pair of oligonucleotide-labelled antibodies and when binding to their correct targets, they give rise to reporter amplicons which are amplified and quantified by microfluidic-based real-time PCR (BioMark™ HD System, Fluidigm Corporation). The data obtained is normalized and used for the relative quantification of the concentration of each analyte [26, 27]. The PEA kits offer the same level of performance as ELISA and comparable sensitivity to standard ELISA kits with much less sample and a higher dynamic range. For a panel description see Additional file 1: Table S1; for the sensitivity and specificity parameters of the PEA analysis see [26, 27].

### Statistics

All statistical analyses were performed on linearized data (linear ddCq) for each analyte. Statistical tests (Mann–Whitney–Wilcoxon test, Benjamini–Hochberg correction, Spearman correlations, Receiver Operating Characteristic (ROC) curve analysis, and Bayesian probability model) were performed using the R statistical software with the Caret package (http://www.r-project.org/; http://topepo.github.io/caret/index.html). The *P* value for each protein was adjusted for multiple comparisons using the False Discovery Rate by the Benjamini–Hochberg procedure. *P*_*corr*_ value < 0.05 was considered significant.

## Results

### Protein pattern of SLE

**Fig. 1:**
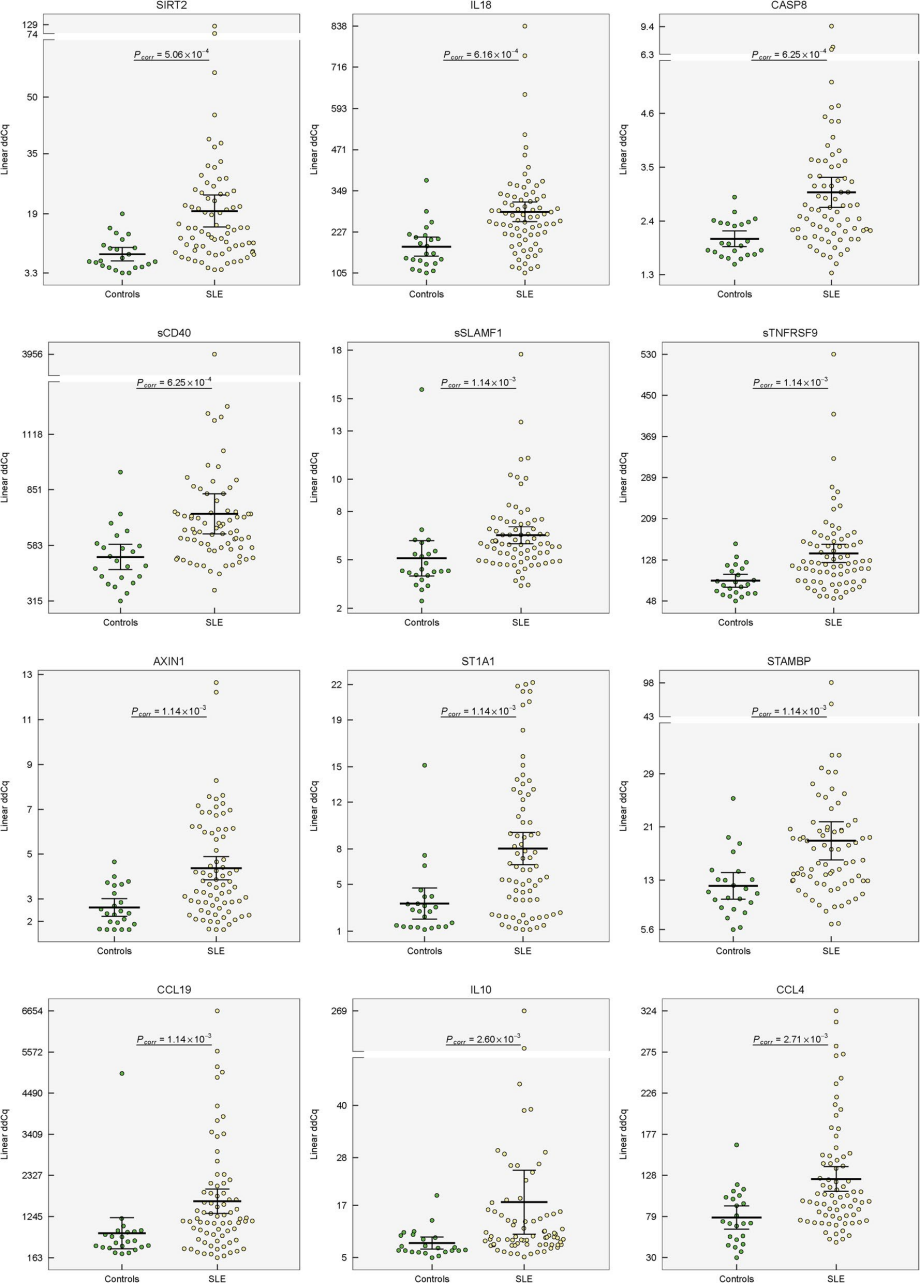
Distribution of serum levels for top-deregulated proteins between healthy controls and SLE. Group means are indicated by horizontal bars, error bars indicate 95% CI; *P*_*corr*_ values after multiple corrections are stated

**Fig. 2:**
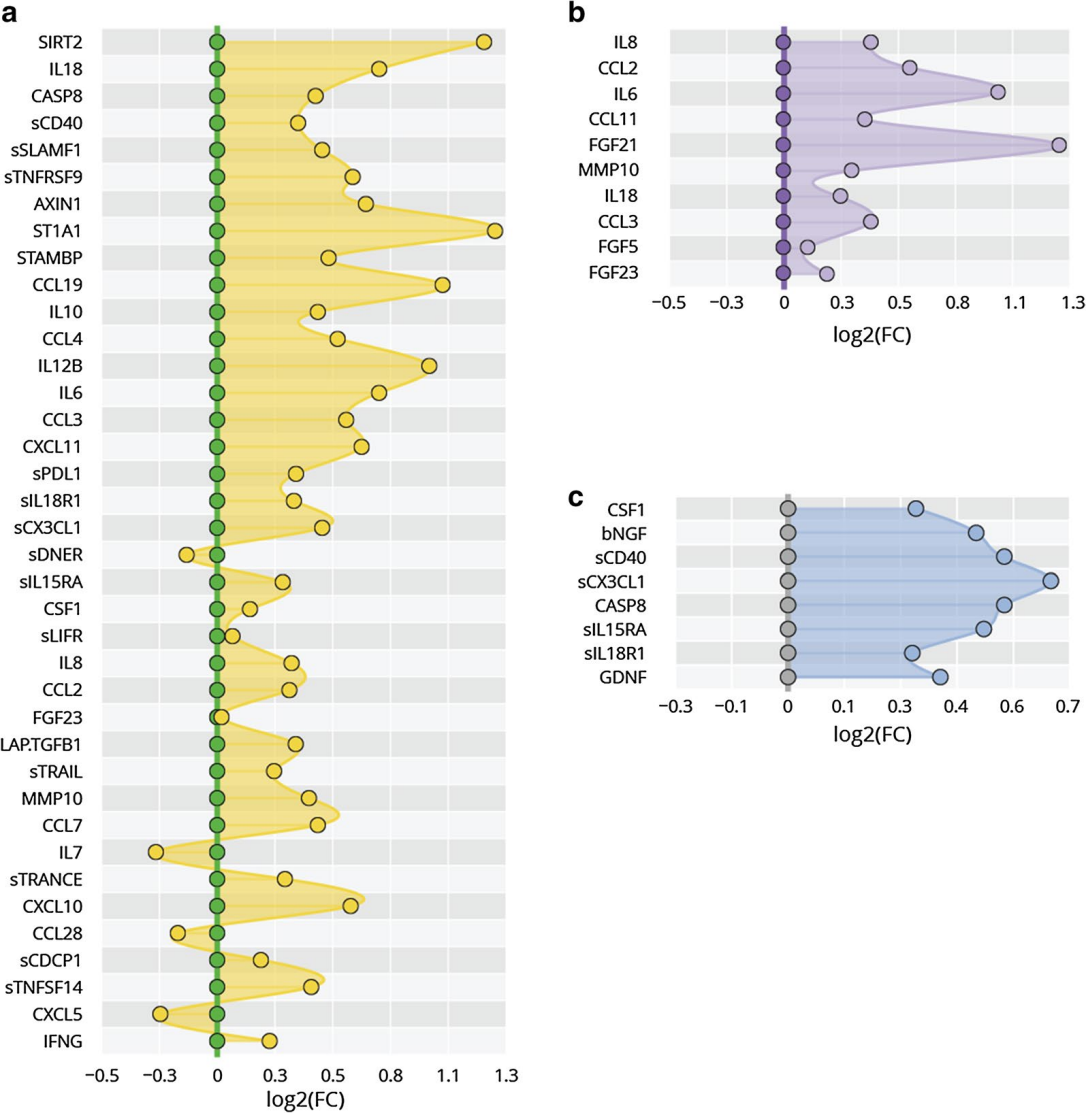
Protein serum fingerprints associated with **a** SLE, **b** organ damage, and **c** active lupus nephritis (LN). Fingerprints are presented as FC (foldchange of group medians) of serum levels of all deregulated serum proteins between particular groups (*P*_*corr*_ < 0.05)

**Fig. 3:**
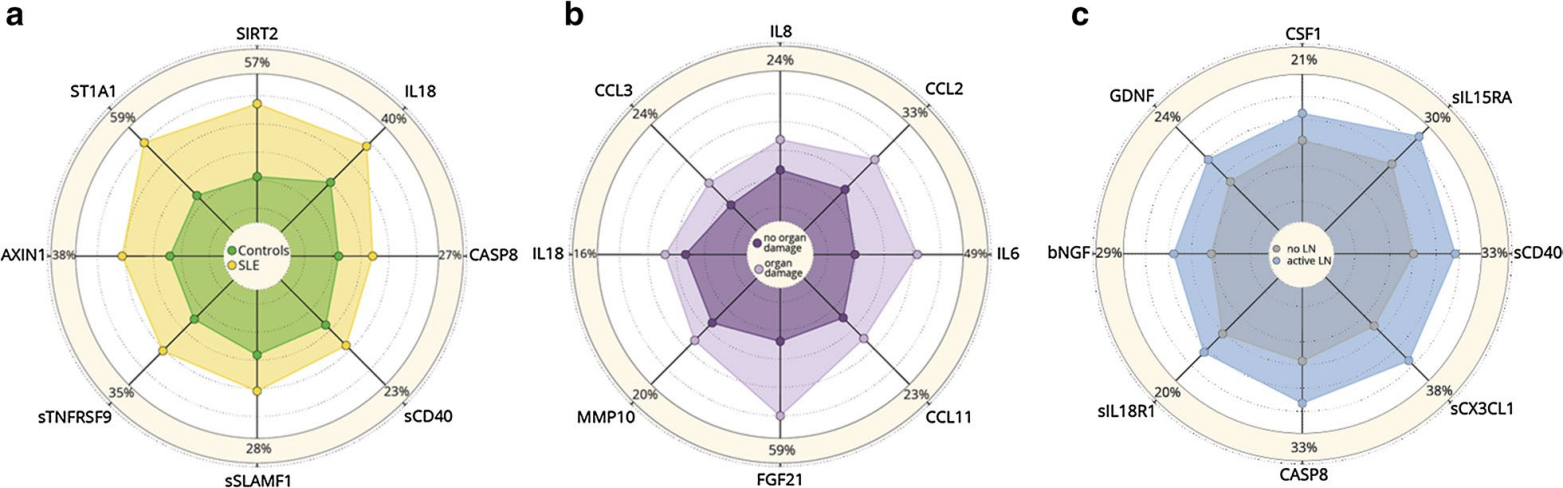
Changes in protein levels for top-deregulated analytes between **a** SLE and controls, **b** patients with/without organ damage, and **c** patients with active lupus nephritis and without lupus nephritis (no LN). Changes are presented as percentage of changes between group medians of particular groups

 In order to assess the serum protein fingerprint associated with SLE, we compared the serum protein levels obtained by PEA immunoassay in the SLE patients and healthy controls. Of 92 biomarkers that were analyzed, the levels of 14 analytes (IL1A, IL2, sIL2RB, IL4, IL5, IL13, IL20, sIL20RA, IL33, TSLP, ARTN, TNF, LIF, NRTN) were below the limit of detection in our sample set and therefore they were excluded from further analysis. Comparing SLE and the controls, 29 proteins were upregulated and sDNER downregulated in SLE (*P*_*corr*_ < 0.05; Table 2a, Additional file 1: Table S2). The distribution of the serum levels of top-upregulated proteins (sirtuin 2, IL18, caspase 8, sCD40/sTNFRSF5, sSLAMF1, sTNFRSF9, axin 1, sulfotransferase 1A1, STAMBP, CCL19/MIP-3ß, IL10, and CCL4/MIP-1β; *P*_*corr*_ < 0.003) is shown in Fig. 1. For the serum protein pattern associated with SLE and the changes in protein levels between SLE and the controls for top-deregulated analytes see Figs. 2a and 3a.

**Table 2:** Serum levels of proteins differentiating between **a** healthy controls *vs* SLE, **b** SLE patients with organ damage (SDI ≥ 1) *vs* those without organ damage (SDI = 0), **c** patients with biopsy-proven active lupus nephritis (active LN) *vs* patients without lupus nephritis (no LN), **d** patients with biopsy-proven active lupus nephritis (active LN) *vs* patients with inactive biopsy-proven lupus nephritis (inactive LN)

**a** Healthy controls *vs* SLE					
Analyte	Mean linear ddCq (95% CI)		FC	*P*	*P*_*corr*_
	Healthy controls	SLE			
SIRT2	8.31 (6.49–10.1)	19.8 (15.5–24.0)	2.33	6.5 × 10^−6^	5.1 × 10^−4^
IL18	183 (155–212)	287 (257–316)	1.67	1.6 × 10^−5^	6.2 × 10^−4^
CASP8	2.04 (1.88–2.20)	2.99 (2.68–3.30)	1.37	2.5 × 10^−5^	6.3 × 10^−4^
sCD40	527 (466–588)	735 (639–831)	1.29	3.2 × 10^−5^	6.3 × 10^−4^
sSLAMF1	5.10 (4.0–6.19)	6.52 (6.0–7.05)	1.39	9.0 × 10^−5^	1.1 × 10^−3^
sTNFRSF9	87.8 (75.2–100)	141 (123–159)	1.54	1.1 × 10^−4^	1.1 × 10^−3^
ST1A1	3.36 (2.04–4.69)	8.04 (6.66–9.43)	2.41	1.3 × 10^−4^	1.1 × 10^−3^
STAMBP	12.1 (10.1–14.1)	18.9 (16.1–21.8)	1.42	1.5 × 10^−4^	1.1 × 10^−3^
CCL19	804 (394–1215)	1646 (1326–1966)	2.04	1.5 × 10^−4^	1.1 × 10^−3^
IL10	8.33 (6.94–9.72)	17.8 (10.4–25.1)	1.38	3.7 × 10^−4^	2.6 × 10^−3^
CCL4	77.7 (63.8–91.6)	123 (109–138)	1.46	4.2 × 10^−4^	2.7 × 10^−3^
IL12B	17.3 (13.3–21.4)	29.3 (25.1–33.5)	1.96	5.7 × 10^−4^	3.4 × 10^−3^
IL6	4.18 (3.28–5.09)	26.9 (−0.62–54.4)	1.67	7.0 × 10^−4^	3.9 × 10^−3^
CCL3	9.58 (4.47–14.7)	35.8 (−11.2–82.9)	1.51	7.6 × 10^−4^	4.0 × 10^−3^
CXCL11	187 (142–232)	343 (279–408)	1.58	1.1 × 10^−3^	5.4 × 10^−3^
sPDL1	3.43 (3.19–3.67)	4.24 (3.91–4.57)	1.28	1.2 × 10^−3^	5.4 × 10^−3^
sIL18R1	106 (90.1–122)	139 (126–152)	1.28	1.4 × 10^−3^	6.0 × 10^−3^
sCX3CL1	86.9 (74.8–99.0)	131 (108–153)	1.39	2.2 × 10^−3^	9.0 × 10^−3^
sDNER	170 (161–179)	150 (143–157)	0.91	2.6 × 10^−3^	1.0 × 10^−2^
sIL15RA	1.90 (1.68–2.13)	2.43 (2.19–2.66)	1.23	2.9 × 10^−3^	1.1 × 10^−2^
CSF1	241 (223–259)	281 (270–293)	1.11	3.3 × 10^−3^	1.2 × 10^−2^
sLIFR	8.35 (7.84–8.85)	10.3 (8.94–11.6)	1.04	3.4 × 10^−3^	1.2 × 10^−2^
IL8	236 (199–273)	383 (281–485)	1.27	3.9 × 10^−3^	1.2 × 10^−2^
CCL2	2133 (1801–2465)	3054 (2660–3449)	1.26	3.9 × 10^−3^	1.2 × 10^−2^
FGF23	2.77 (2.63–2.91)	4.32 (2.95–5.70)	1.01	5.8 × 10^−3^	1.8 × 10^−2^
LAP.TGFB1	131 (117–146)	158 (148–167)	1.28	6.6 × 10^−3^	1.9 × 10^−2^
sTRAIL	517 (470–565)	602 (568–636)	1.20	1.1 × 10^−2^	3.1 × 10^−2^
MMP10	98.5 (81.5–116)	156 (128–184)	1.34	1.4 × 10^−2^	3.7 × 10^−2^
CCL7	5.70 (4.57–6.83)	12.5 (7.31–17.7)	1.38	1.6 × 10^−2^	4.1 × 10^−2^

**b** SDI = 0 *vs* SDI ≥ 1					
Analyte	Mean linear ddCq (95% CI)		FC	*P*	*P*_*corr*_
	SDI = 0	SDI ≥ 1			
IL8	286 (215–358)	459 (286–632)	1.32	2.7 × 10^−5^	2.1 × 10^−3^
CCL2	2485 (2011–2960)	3502 (2924–4080)	1.50	4.3 × 10^−4^	1.6 × 10^−2^
IL6	8.28 (3.95–12.6)	41.5 (−7.95–91.0)	1.98	6.5 × 10^−4^	1.6 × 10^−2^
CCL11	273 (247–299)	344 (315–373)	1.30	8.0 × 10^−4^	1.6 × 10^−2^
FGF21	56.1 (28.8–83.4)	257 (57.1–456)	2.43	1.0 × 10^−3^	1.6 × 10^−2^
MMP10	112 (93.4–131)	190 (144–237)	1.24	2.4 × 10^−3^	3.1 × 10^−2^
IL18	255 (208–302)	311 (274–349)	1.19	3.7 × 10^−3^	4.1 × 10^−2^
CCL3	9.70 (8.24–11.2)	56.4 (−28.7–142)	1.32	4.8 × 10^−3^	4.4 × 10^−2^
FGF5	2.25 (2.13–2.37)	2.72 (2.31–3.12)	1.08	5.1 × 10^−3^	4.4 × 10^−2^
FGF23	3.75 (2.51–5.00)	4.77 (2.47–7.06)	1.15	5.7 × 10^−3^	4.4 × 10^−2^

**c** No LN *vs* active LN					
Analyte	Mean linear ddCq (95% CI)		FC	*P*	*P*_*corr*_
	No LN	Active LN			
CSF1	266 (254–278)	340 (310–370)	1.27	4.0 × 10^−5^	2.7 × 10^−3^
sIL15RA	2.17 (2.03–2.32)	3.65 (2.57–4.73)	1.43	9.0 × 10^−5^	2.7 × 10^−3^
sCD40	645 (595–695)	1116 (587–1645)	1.48	1.0 × 10^−4^	2.7 × 10^−3^
sCX3CL1	103 (93.2–112)	247 (134–359)	1.62	2.8 × 10^−4^	5.4 × 10^−3^
CASP8	2.80 (2.39–3.21)	3.84 (3.06–4.73)	1.49	1.1 × 10^−3^	1.7 × 10^−2^
sIL18R1	129 (119–139)	186 (121–251)	1.26	1.9 × 10^−3^	2.3 × 10^−2^
bNGF	2.66 (2.47–2.84)	3.66 (2.96–4.36)	1.41	2.2 × 10^−3^	2.3 × 10^−2^
GDNF	4.66 (4.37–4.96)	6.16 (4.98–7.33)	1.32	2.3 × 10^−3^	2.3 × 10^−2^

**d** Inactive LN *vs* active LN					
Analyte	Mean linear ddCq (95% CI)		FC	*P*	*P*_*corr*_
	Inactive LN	Active LN			
sIL15RA	2.15 (1.82–2.48)	3.65 (2.57–4.73)	1.56	8.9 × 10^−4^	6.9 × 10^−2^
CSF1	280 (253–306)	340 (310–370)	1.28	4.7 × 10^−3^	0.15
bNGF	2.64 (2.43–2.85)	3.66 (2.96–4.36)	1.29	4.7 × 10^−3^	0.15
sIL18R1	73.4 (66.2–80.5)	186 (121–251)	1.31	6.6 × 10^−3^	0.15
sCD40	688 (636–740)	1116 (587–1645)	1.32	1.2 × 10^−2^	0.18
sCX3CL1	118 (93.6–143)	247 (134–359)	1.42	2.3 × 10^−2^	0.25
CASP8	2.84 (2.42–3.26)	3.84 (3.06–4.73)	1.33	2.3 × 10^−2^	0.25

*P*_*corr*_ value corrected for multiple comparisons (Benjamini–Hochberg correction)

FC (fold-change) between group medians of linear ddCq

Because of the suggested central role of the IFN pathway in SLE pathogenesis by promoting feedback loops progressively disrupting peripheral immune tolerance and driving disease activity [28, 29], we investigated the IFN protein “signature” of nine IFN-regulated cytokines. Because of the reported association of an increased IFN gene expression “signature” with disease activity [28, 29], we performed correlation analysis among the protein levels of IFN-regulated chemokines and disease activity as assessed by SLEDAI. The analysis revealed elevation of six IFN-regulated cytokines (IL6, CCL2/MCP1, CCL3/MIP-1α, sCD40, CXCL11, and CCL19; *P*_*corr*_ ≤ 0.01) in SLE and three (CCL8/MCP2, CXCL9, and CXCL10) did not reach significance (*P*_*corr*_ > 0.05). Interestingly, only a mild positive correlation (r = 0.25, *P* = 0.03; Additional file 1: Table S3) was observed between the levels of IFN-regulated chemokines and disease activity as assessed by SLEDAI. Disease activity assessed by SLEDAI correlated better with the following analytes: IL8, GDNF, CX3CL1/fractalkine (r ≥ 0.403, *P* ≤ 0.0003), and CCL7/MCP3, IL15RA, VEGFA, and MMP10 (r ≥ 0.355, *P* ≤ 0.002; Additional file 1: Table S3).

### Protein pattern of organ damage

To obtain the protein pattern associated with organ damage, we compared the serum patterns from SLE patients with/without organ damage and subgroups according to the SDI (SDI ≥ 2/SDI = 1/SDI = 0).

**Fig. 4:**
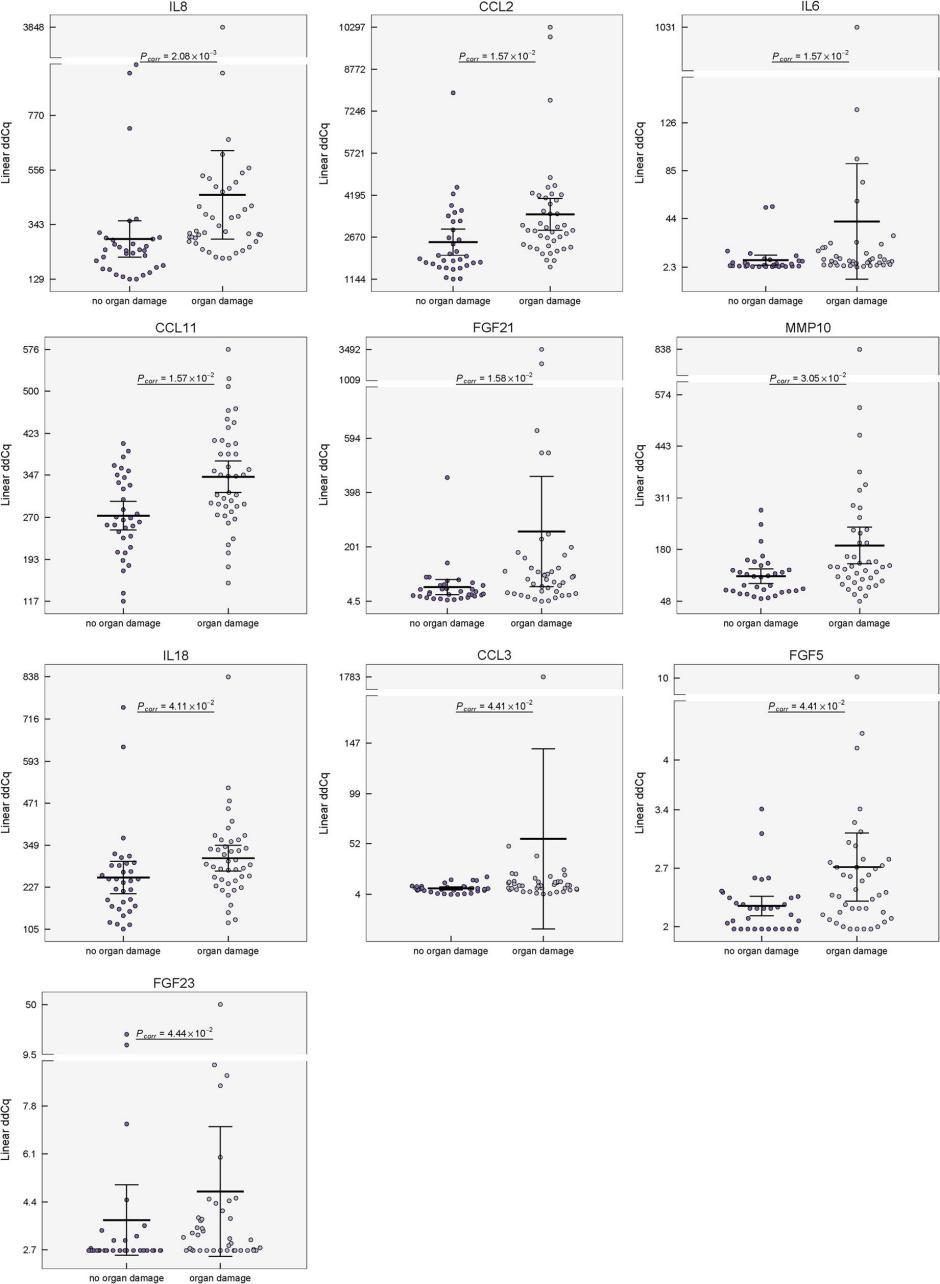
Distribution of serum levels of proteins distinguishing SLE patients with/without organ damage. Group means are indicated by horizontal bars, error bars indicate 95% CI; *P*_*corr*_ values for differences after multiple corrections are stated

 The distribution of damaged organs in our patient group and reported cohorts is shown in Additional file 1: Figure S1, Table S4. In the patients with organ damage (SDI ≥ 1), elevated serum levels of IL8, CCL2, IL6, CCL11/eotaxin, FGF21, MMP10, IL18, CCL3, FGF5, and FGF23 (*P*_*corr*_ < 0.05) were detected (Table 2b, Fig. 4). The serum protein pattern associated with organ damage and the changes in protein levels between SLE patients with/without organ damage are shown in Figs. 2b and 3b. Although the serum level of CCL11 did not differ between the controls and SLE patients as a whole, the patients with organ damage had higher levels of CCL11 in comparison to those with no organ damage, as well as to the control group (Additional file 1: Figure S2a). We did not observe differences in serum protein pattern between patients with SDI = 1 and SDI ≥ 2 (data not shown).

Among organ damage associated analytes, the cumulative dose of glucocorticoids correlated positively with levels of IL8, CCL11 (r ≥ 0.326, *P* ≤ 0.004), CCL2 and MMP10 (r ≥ 0.249, *P* < 0.05; Additional file 1: Table S3). Additionaly, cumulative dose of glucocorticoids correlated with BDNF, CCL25, CXCL1, GDNF, IL17C, sADA, sCDCP1, sIL18R1, sSCF, and sTGFA (*P* < 0.05; Additional file 1: Table S3). Moreover, IL8 (r = 0.416, *P* = 0.0002), MMP10 (r = 0.355, *P* = 0.002), CCL2, and CCL11 (r ≥ 0.261, *P* ≤ 0.02; Additional file 1: Table S3) correlated positively with disease activity. In line with other reports, a higher cumulative dosage of glucocorticoids was registered in the patients with SDI ≥ 1 (mean of 30.6 g, min–max 2.6–79.2 g) compared with those without damage (12.8, 0–54.0). Regarding association of disease duration and serum levels of studied proteins, we observed only mild association for CCL11 (r = 0.230, *P* = 0.047). The disease duration in SLE patients correlated with SDI (r = 0.298, *P* = 0.009).

### Protein pattern of active lupus nephritis and other clinical subsets of SLE

To investigate the serum patterns associated with active LN, we compared subgroups of SLE patients with/without biopsy-proven LN and subgroups of patients with LN classified by the renal SLEDAI as active (renal SLEDAI ≥ 4) or inactive renal disease at the day of sampling. Moreover, we assessed serum patterns associated with other clinical subsets of SLE as neurological, hematological, cardiovascular, skin and musculoskeletal involvements, antiphospholipid syndrome, and renal disorder.

**Fig. 5:**
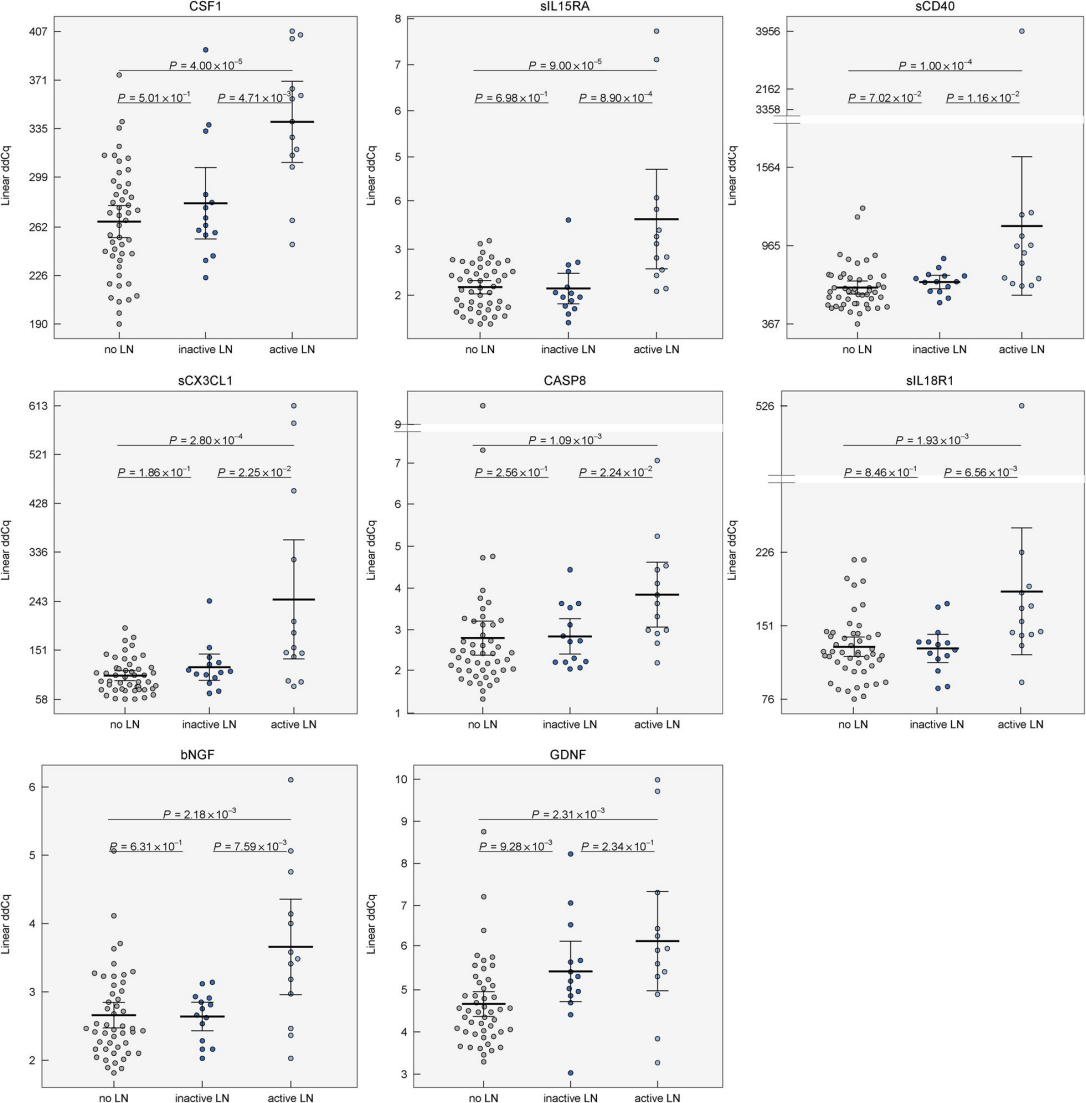
Distribution of serum levels of proteins distinguishing SLE patients without lupus nephritis (LN), with inactive lupus nephritis (inactive LN) and active lupus nephritis (active LN). Group means are indicated by horizontal bars, error bars indicate 95% CI

The analysis in biopsy-proven LN patients with active renal disease revealed elevated protein levels of CSF1, sIL15RA, sCD40, sCX3CL1, caspase 8, sIL18R1, bNGF, and GDNF compared to those without LN (Table 2c, Fig. 5). Although the serum levels of GDNF did not differ between the control group and SLE as a whole, its level was enhanced in the patients with LN in comparison to those without LN and the control group (Additional file 1: Figure S2b). The serum protein pattern associated with active LN and the changes in protein levels between the SLE patients without LN and active LN are shown in Figs. 2c and 3c.

When LN patients with active renal disease was compared to inactive LN subgroup, elevation of sIL15RA, CSF1, bNGF, sIL18R1, sCD40, sCX3CL1, and caspase 8 (*P* < 0.05, Table 2d, Fig. 5), but not GDNF, in active LN patients was observed.

In the other studied clinical subsets no differences in the serum pattern were detected. The subanalysis confirmed that no candidate biomarker for SLE, organ damage and/or LN are influenced by the gender (data not shown).

### Identification of patients with a high probability of organ damage and active lupus nephritis

To investigate the utility of the serum levels of phenotype-associated proteins for the identification of patients with a high probability of severe phenotypes, we constructed probability plots for phenotype-associated proteins based on a Bayesian statistical approach. Additionally, we constructed ROC curves for the proteins associated with organ damage and active LN.

**Fig. 6:**
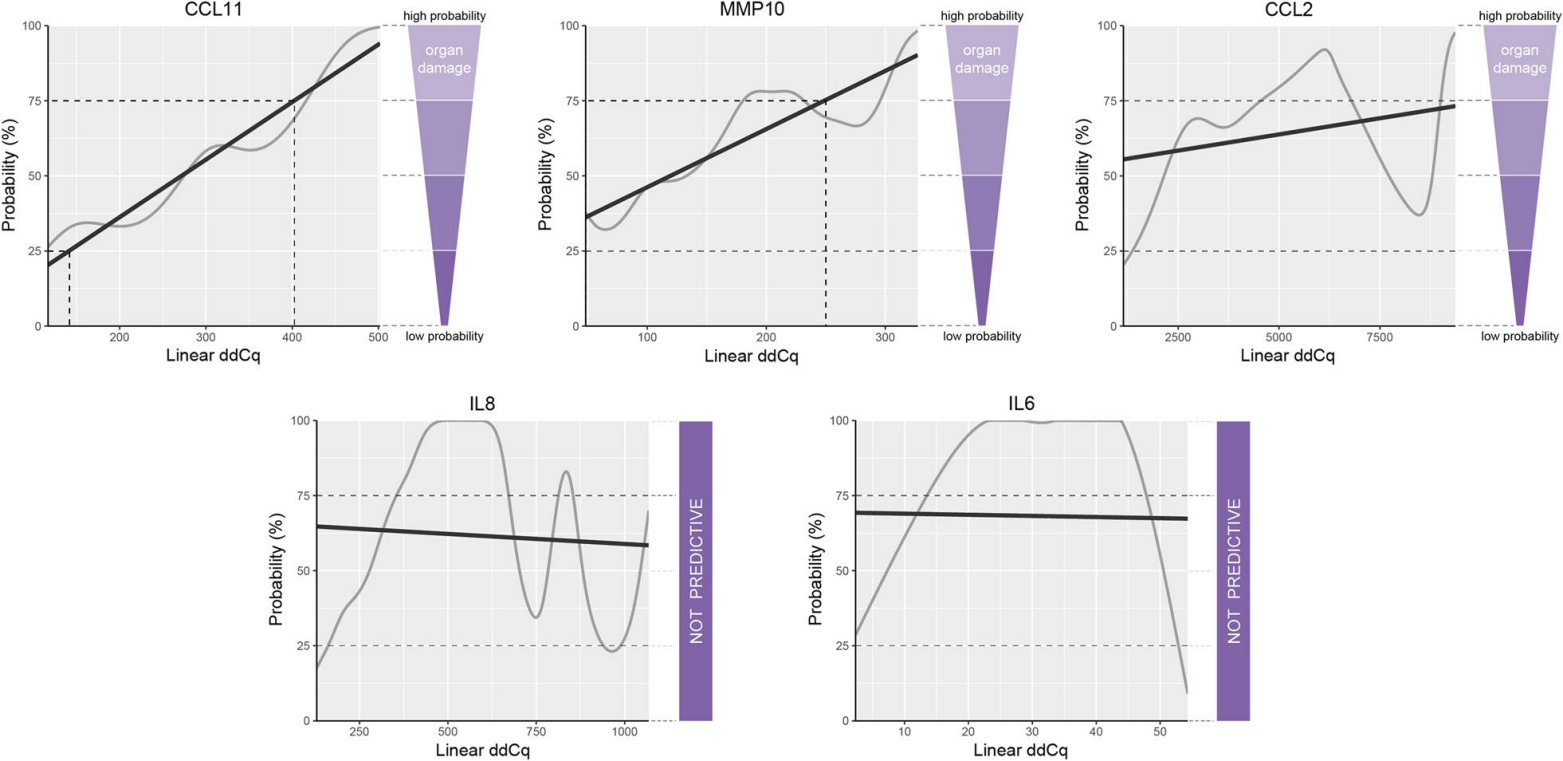
Probability plots of serum analytes associated with organ damage in SLE patients. The grey curve represents a simulated model based on the individual patient serum levels and the black line represents overall trend calculated by the Bayesian statistical approach. The increasing overall trend the higher probability of organ damage. Higher serum levels of CCL11 and MMP10 correspond to higher probability of organ damage, lower serum levels of these analytes to lower probability of organ damage. IL8 and IL6 serum levels were not informative for organ damage prediction

In organ damage, the best predictive model was observed for the serum levels of CCL11 and MMP10, followed by CCL2, whereas IL6 and IL8 were not informative (Fig. 6). Higher serum levels of CCL11 and MMP10 correspond to a higher probability of organ damage. For the analytes associated with organ damage, the ROC curve analysis showed that the area under the curve (AUC) of IL8, CCL2, IL6, CCL11, FGF21, MMP10, IL18, CCL3, FGF5, and FGF23 was 0.784, 0.738, 0.731, 0.727, 0.723, 0.706, 0.697, 0.691, 0.689, and 0.676, respectively (Additional file 1: Figure S3a; for sensitivity, specificity, and other parameters see Additional file 1: Table S5a).

**Fig. 7:**
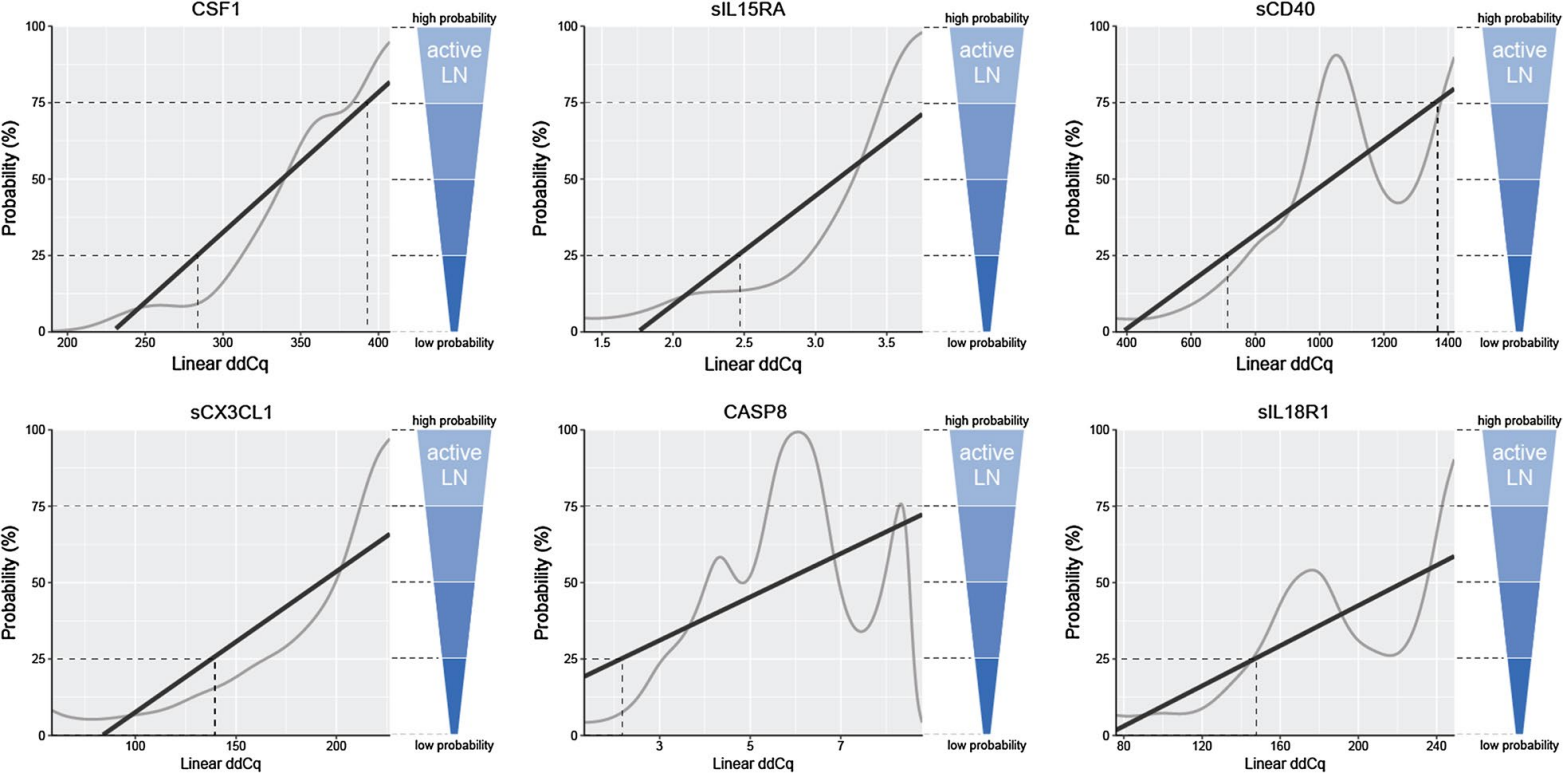
Probability plots of serum analytes associated with active lupus nephritis (LN) in SLE patients. The grey curve represents a simulated model based on the individual patient serum levels and the black line represents overall trend calculated by the Bayesian statistical approach. The increasing overall trend the higher probability of active LN. Higher serum levels correspond to higher probability of active LN, lower serum levels of these analytes to lower probability of active LN. The best predictive value was observed for CSF1, sIL15RA, sCD40, sCX3CL1, caspase 8 (CASP8), and sIL18R1

In active LN, the best predictive value was observed for CSF1, sIL15RA, sCD40, sCX3CL1, caspase 8, and sIL18R1 (Fig. 7). Higher serum levels of all these analytes correspond to a higher probability of the presence of active LN. For the analytes associated with active LN, the ROC curve analysis showed that the AUC of CSF1, sIL15RA, sCD40, sCX3CL1, caspase 8, sIL18R1, bNGF, and GDNF were 0.873, 0.857, 0.854, 0.832, 0.798, 0.783, 0.780, and 0.778, respectively (Additional file 1: Figure S3b, Table S5b). Moreover, we observed great sensitivity and specificity for proteins sIL15RA (AUC: 0.879, sensitivity: 100%, specificity: 64.3%), CSF1 (0.813, 84.6, 78.6), sIL18R1 (0.810, 84.6, 78.6), and bNGF (0.805, 69.2, 100) showing good discrimination between active and inactive renal disease in LN patient subgroup (Additional file 1: Figure S3c, Table S5c). Inactive LN patients do not differ from patients without LN, except for GDNF (Fig. 5), suggesting that serum GDNF level remains elevated even when LN is inactive.

All nominated biomarkers associated with organ damage and active LN showed better discrimination ability in our cohort than the classical markers (Additional file 1: Table S6). The only exception was proteinuria (AUC 0.869), one of the criteria for renal SLEDAI classification.

## Discussion

Using innovative highly sensitive multiplex PEA analysis on 92 inflammation-related proteins, we identified the serum protein pattern associated with SLE, with many proteins not yet reported in this disease. Moreover, we identified the serum patterns associated with irreversible organ damage and active LN and identified proteins showing utility for the identification of patients at risk of these severe disease manifestations.

This serum protein study in SLE patients revealed the deregulation of 30 proteins in SLE. The majority of the upregulated proteins were known inflammatory mediators: IL6, IL10 [30], IL18 [31], CX3CL1 [32], CCL2 [33], CCL3, CCL7, CCL19 [34], and FGF23 [35] already reported in SLE previously. Interestingly, the most upregulated proteins—sirtuin 2 and caspase 8—were not associated with SLE or even with any autoimmune disease. However, recent reports in animal models and cell lines support their involvement in inflammation and autoimmunity. Regarding sirtuin 2, macrophages expressing this protein produced more iNOS/NO upon LPS stimulation than those with depleted sirtuin 2 [36]. This result was also confirmed in vivo, where WT mice responded to LPS by increased NO levels and a higher amount of M1-macrophages compared to sirtuin 2 KO mice [36]. Elevated sirtuin 2 also contributed to prolonged hypoinflammation in a septic murine model [37]. Regarding caspase 8, a protein widely recognized for its role in apoptosis, recent reports identify this enzyme as a crucial regulator of inflammation through NFκB activation and cleavage of pro-IL1β and/or pro-IL18, similarly to caspase 1 [38, 39]. These observations lead us to suggest that caspase 8 may also promote autoimmunity by stimulating IL17 production by T cells, as shown for caspase 1 [40]. Moreover, the therapeutic potential of caspase 8 is supported by the observation of attenuated retinal ischemic damage resulting from the inhibition of caspase 8, resulting in the blockade of IL1β production [41]. However, there is evidence about the pleiotropic effects of sirtuin 2 and caspase 8, and thus future studies on their role in SLE are needed.

Further highly upregulated proteins, IL18 and sulfotransferase 1A1, were already reported in autoimmunity. An elevated IL18 serum level was reported in SLE [42], especially in LN patients [43, 44]. Regarding sulfotransferase 1A1, higher activity was found in autoimmune thyroid disease glands compared to normal thyroids [45], but no information yet exists in SLE. Interestingly, we did not detect any elevation of the serum level of the previously reported SLE-associated factor TWEAK and IFNγ [46, 47]. Despite the reported association of the IFN gene expression “signature” with disease activity in SLE [28, 29], we did not confirm either elevated levels of the IFN-regulated chemokines CCL8, CXCL9, CXCL10 or strong correlation of the IFN protein “signature” with disease activity at the protein level in the sera of our patients. Our observation is in line with others [28], thus supporting the opinion that cytokine levels in serum are a less sensitive readout for activation of the IFN pathway than the gene expression “signature”.

Despite tremendous efforts, the greatest challenges still remain in the management of SLE patients with severe organ damage and active LN. Thus, there is a need to identify novel biomarkers that will better facilitate the assessment of organ involvement and disease activity. In our study, SLE patients with organ damage had elevated serum levels of IL8, CCL2, IL6, CCL11, FGF21, MMP10, IL18, CCL3, FGF5, and FGF23 compared to those without organ damage. Of these, enhanced levels of CCL11, MMP10, and CCL2 were informative for the identification of patients with organ damage. Importantly, CCL11, MMP10, and CCL2 also correlated with disease activity. The elevation of the chemokine CCL11 was already associated with damage to various organs, as shown in idiopathic retroperitoneal fibrosis [48] and liver cirrhosis patients [49]. Moreover, in murine models of lung fibrosis [50], as well as of eosinophilic myocarditis [51], the blockade of the CCL11-CCR3 pathway prevented organ damage. Similarly, MMP10 was linked to renal damage [52] and tissue destruction in arthritis [53]. Elevation of MMP10 was already reported in SLE patients [54] and in a murine LN model with glomerulonephritis [55]. Another protein associated with organ damage, CCL2, was already reported in kidney damage in lupus murine models [56] and in SLE patients with irreversible renal damage [57]. Although IL6 and IL8, cytokines involved in the pathogenesis of SLE, were also enhanced in our patients with organ damage, our analysis did not support their predictive value for this severe phenotype. The usefulness of CCL11, MMP10, and CCL2 as biomarkers or possible treatment targets needs to be elucidated in future studies.

Lupus nephritis is considered another challenging SLE phenotype from the point of view of its prediction and preemptive diagnostics. Renal biopsy is still the gold standard to assess the renal involvement of SLE and its severity and pathological category [8]. The search for non-invasive biomarkers in serum and urine reflecting the renal disease activity is therefore a major focus of interest. Our serum protein analysis in LN patients with active renal disease revealed upregulated levels of CSF1, sIL15RA, sCD40, sCX3CL1, caspase 8, sIL18R1, bNGF, and GDNF compared to those without LN. All these markers showed excellent discrimination for active LN, significantly better than the classical markers as shown by us and others [9, 10]. Moreover, we observed good discrimination between active and inactive renal disease in LN patient subgroup for all markers, except for GDNF. Apart from caspase 8 and sIL15RA, emerging evidence of the active involvement of these proteins in LN already exists. Regarding CSF1, elevated serum levels in patients with SLE were shown to reflect kidney histopathology and to predict renal disease activity [58]. Moreover, CSF1 deficiency protected against LN in murine models [59]. Enhanced protein and gene expression of IL15RA was detected in leucocytes from SLE patients [60, 61], probably as a results of hydroxymethylation in promoter region of this gene in SLE [61]. There is also evidence about the crucial role of the CD40-CD40L system in the development, progression and outcome of SLE [62]. Enhanced CD40L protein level was detected in sera from SLE patients [62, 63] as well as class III and IV LN and other inflammatory renal diseases [64]. Moreover, CD40 gene silencing reduced the progression of experimental LN [65]. Regarding sCX3CL1, elevated expression was reported in proliferative LN [66] and the administration of a CX3CL1 antagonist to mice delayed the initiation and ameliorated the progression of LN [67]. Also enhanced expression of IL18R1 has already been reported in SLE patients [68] as well as in peripheral plasmacytoid DCs in active LN patients [69]. Similarly, increased levels of NGF, a complex of 3 subunits—aNGF, bNGF, and gNGF, has been reported in the sera of SLE patients [70] and various renal disorders [71]. Regarding GDNF, a high expression of this protein was detected in renal biopsies from patients with proteinuric nephropathy [72] and increased plasma levels of GDNF were reported in patients with chronic renal diseases [73]. This mesangial autocrine growth factor was shown to play a pivotal role in mesangial cell proliferation, which is essential for the progression of various glomerular diseases [74]. Our study did not confirm IL18 as a useful biomarker to assess the activity of renal disease, as reported by others [42, 43]. On the other hand, our results nominated spectrum of novel biomarkers of renal involvement for further confirmation studies.

Althougth relatively high sensitivity and specificity was obtained for each individual marker in our LN and organ damage subgroups, we believe that using rather a panel of multiple biomarkers and/or combination with other clinical and laboratory parameters would be an appropriate approach in the identification of patients with these severe manifestations.

## Conclusions

This exploratory study revealed many novel proteins associated with SLE for future immunopathogenesis studies, as well as nominating candidate biomarkers for irreversible organ damage and active lupus nephritis. Future studies on larger cohorts with well-defined phenotypes as well as the longitudinal follow-up during disease development are needed to prove the suitability of these proteins or their combinations as biomarkers for organ damage and lupus nephritis, with special emphasis on disease activity.

## Additional file

**Additional file 1.** SLE serum pattern: Supplemental figures and tables.

## Abbreviations

4E-BP1: eukaryotic translation initiation factor 4E-binding protein 1
aNGF: alpha-nerve growth factor
APRIL: a proliferation-inducing ligand
ARTN: artemin
AXIN1: axin 1
BLyS: B lymphocyte stimulator
BDNF: brain-derived neurotrophic factor
bNGF: beta-nerve growth factor
CASP8: caspase 8
CCL2: C-C motif chemokine ligand 2, monocyte chemotactic protein 1
CCL3: C-C motif chemokine ligand 3, macrophage inflammatory protein 1-alpha
CCL4: C-C motif chemokine ligand 4, macrophage inflammatory protein 1-beta
CCL7: C-C motif chemokine ligand 7, monocyte chemotactic protein 3
CCL8: C-C motif chemokine ligand 8, monocyte chemotactic protein 2
CCL11: C-C motif chemokine ligand 11, eotaxin-1
CCL13: C-C motif chemokine ligand 13, monocyte chemotactic protein 4
CCL19: C-C motif chemokine ligand 19, macrophage inflammatory protein 3-beta
CCL20: C-C motif chemokine ligand 20, macrophage inflammatory protein 3-alpha
CCL23: C-C motif chemokine ligand 23, macrophage inflammatory protein 3
CCL25: C-C motif chemokine ligand 25
CCL28: C-C motif chemokine ligand 28
CD40L: cluster of differentiation 40 ligand
CCR3: C-C chemokine receptor type 3
CSF1: macrophage colony-stimulating factor 1
CST5: cystatin D
CXCL1: C-X-C motif chemokine ligand 1
CXCL5: C-X-C motif chemokine ligand 5
CXCL6: C-X-C motif chemokine ligand 6
CXCL9: C-X-C motif chemokine ligand 9
CXCL10: C-X-C motif chemokine ligand 10
CXCL11: C-X-C motif chemokine ligand 11
EN.RAGE: extracellular newly identified receptor for advanced glycation end-products binding protein, protein S100-A12
FGF5: fibroblast growth factor 5
FGF19: fibroblast growth factor 19
FGF21: fibroblast growth factor 21
FGF23: fibroblast growth factor 23
gNGF: gamma-nerve growth factor
GDNF: glial cell line-derived neurotrophic factor
IFN: interferon
IFNG: interferon gamma
IGFBP2: insulin like growth factor binding protein 2
IL1A: interleukin-1 alpha
IL1B: interleukin-1 beta
IL2: interleukin-2
IL4: interleukin-4
IL5: interleukin-5
IL6: interleukin-6
IL7: interleukin-7
IL8: interleukin-8
IL10: interleukin-10
IL12B: interleukin-12 beta
IL13: interleukin-13
IL17: interleukin-17
IL17A: interleukin-17A
IL17C: interleukin-17C
IL18: interleukin-18
IL20: interleukin-20
IL24: interleukin-24
IL33: interleukin-33
iNOS/NO: inducible nitric oxide synthase/nitrogen oxide
KO: knock out
LAP.TGFB1: latency-associated peptide transforming growth factor beta-1
LIF: leukemia inhibitory factor
LN: lupus nephritis
LPS: lipopolysaccharide
MMP1: matrix metalloproteinase-1
MMP10: matrix metalloproteinase-10
NFκB: nuclear factor kappa B
NGF: nerve growth factor
NRTN: neurturin
NT3: neurotrophin-3
OSM: oncostatin-M
PCR: polymerase chain reaction
PEA: proximity extension immunoassay
ROC: receiver operating characteristic
sADA: adenosine deaminase, soluble
sCD244: natural killer cell receptor 2B4, soluble
sCD40: cluster of differentiation 40, tumor necrosis factor receptor superfamily member 5, soluble
sCD5: cluster of differentiation 5, soluble
sCD6: cluster of differentiation 6, soluble
sCDCP1: CUB domain-containing protein 1, soluble
sCX3CL1: C-X3-C motif chemokine ligand 1, fractalkine, soluble
SDI: Systemic Lupus International Collaborating Clinics/American College of Rheumatology Damage Index
sDNER: delta and notch-like epidermal growth factor-related receptor, soluble
sFlt3L: Fms-related tyrosine kinase 3 ligand, soluble
sHGF: hepatocyte growth factor
sIL10RA: interleukin-10 receptor subunit alpha, soluble
sIL10RB: interleukin-10 receptor subunit beta, soluble
sIL15RA: interleukin-15 receptor subunit alpha, soluble
sIL18R1: interleukin-18 receptor 1, soluble
sIL20RA: interleukin-20 receptor subunit alpha, soluble
sIL22RA1: interleukin-22 receptor subunit alpha-1, soluble
sIL2RB: interleukin-2 receptor subunit beta, soluble
SIRT2: sirtuin 2
SLE: systemic lupus erythematosus
SLEDAI: Systemic Lupus Erythematosus Disease Activity Index
sLIFR: leukemia inhibitory factor receptor, soluble
sOPG: osteoprotegerin, soluble
sPDL1: programmed cell death 1 ligand 1, soluble
sSCF: stem cell factor, soluble
sSLAMF1: signaling lymphocytic activation molecule 1, soluble
ST1A1: sulfotransferase 1A1
STAMBP: signal transducing adaptor molecule-binding protein
sTGFA: transforming growth factor alpha, soluble
sTNFB: tumor necrosis factor-beta, soluble
sTNFRSF9: tumor necrosis factor receptor superfamily member 9, soluble
sTNFSF14: tumor necrosis factor ligand superfamily member 14, soluble
sTRAIL: tumor necrosis factor-related apoptosis-inducing ligand, soluble
sTRANCE: tumor necrosis factor-related activation-induced cytokine, soluble
sTWEAK: tumor necrosis factor ligand superfamily member 12, soluble
TGFβ: transforming growth factor beta
TNF: tumor necrosis factor
TSLP: thymic stromal lymphopoietin
uPA: urokinase-type plasminogen activator
VEGFA: vascular endothelial growth factor A
WT: wild type
