# Supplementary material for: Serum protein pattern associated with organ damage and lupus nephritis in systemic lupus erythematosus revealed by PEA immunoassay
Source: Clin Proteomics. 2017 Oct 3;14:32. doi: 10.1186/s12014-017-9167-8 (PMC5627398; doi:10.1186/s12014-017-9167-8)
Supplement: Supplementary file 1 — Additional file 1. SLE serum pattern: Supplemental figures and tables. [file 12014_2017_9167_MOESM1_ESM.pdf]

ADDITIONAL FILE 1

Serum protein pattern associated with organ damage and lupus nephritis in systemic lupus erythematosus revealed by PEA immunoassay

Anna Petrackova, Andrea Smrzova, Petr Gajdos, Marketa Schubertova, Petra Schneiderova, Pavel Kromer, Vaclav Snasel, Martina Skacelova, Frantisek Mrazek, Josef Zadrazil, Pavel Horak, Eva Kriegova

Table S1 List of investigated proteins.

Protein symbol	Protein name
4E-BP1	Eukaryotic translation initiation factor 4E-binding protein 1
ARTN	Artemin
AXIN1	Axin 1
BDNF	Brain-derived neurotrophic factor
bNGF	Beta-nerve growth factor
CASP8	Caspase 8
CCL2	C-C motif chemokine ligand 2, monocyte chemotactic protein 1
CCL3	C-C motif chemokine ligand 3, macrophage inflammatory protein 1-alpha
CCL4	C-C motif chemokine ligand 4, macrophage inflammatory protein 1-beta
CCL7	C-C motif chemokine ligand 7, monocyte chemotactic protein 3
CCL8	C-C motif chemokine ligand 8, monocyte chemotactic protein 2
CCL11	C-C motif chemokine ligand 11, eotaxin-1
CCL13	C-C motif chemokine ligand 13, monocyte chemotactic protein 4
CCL19	C-C motif chemokine ligand 19, macrophage inflammatory protein 3-beta
CCL20	C-C motif chemokine ligand 20, macrophage inflammatory protein 3-alpha
CCL23	C-C motif chemokine ligand 23, macrophage inflammatory protein 3
CCL25	C-C motif chemokine ligand 25
CCL28	C-C motif chemokine ligand 28
CSF1	Macrophage colony-stimulating factor 1
CST5	Cystatin D
CXCL1	C-X-C motif chemokine ligand 1
CXCL5	C-X-C motif chemokine ligand 5
CXCL6	C-X-C motif chemokine ligand 6
CXCL9	C-X-C motif chemokine ligand 9
CXCL10	C-X-C motif chemokine ligand 10
CXCL11	C-X-C motif chemokine ligand 11
EN.RAGE	Extracellular newly identified receptor for advanced glycation end-products binding protein, protein S100-A12
FGF5	Fibroblast growth factor 5
FGF19	Fibroblast growth factor 19

FGF21	Fibroblast growth factor 21
FGF23	Fibroblast growth factor 23
GDNF	Glial cell line-derived neurotrophic factor
IFNG	Interferon gamma
IL1A	Interleukin-1 alpha
IL2	Interleukin-2
IL4	Interleukin-4
IL5	Interleukin-5
IL6	Interleukin-6
IL7	Interleukin-7
IL8	Interleukin-8
IL10	Interleukin-10
IL12B	Interleukin-12 beta
IL13	Interleukin-13
IL17A	Interleukin-17A
IL17C	Interleukin-17C
IL18	Interleukin-18
IL20	Interleukin-20
IL24	Interleukin-24
IL33	Interleukin-33
LAP.TGFB1	Latency-associated peptide transforming growth factor beta-1
LIF	Leukemia inhibitory factor
MMP1	Matrix metalloproteinase-1
MMP10	Matrix metalloproteinase-10
NRTN	Neurturin
NT3	Neurotrophin-3
OSM	Oncostatin-M
sADA	Adenosine deaminase, soluble
sCD5	Cluster of differentiation 5, soluble
sCD6	Cluster of differentiation 6, soluble
sCD40	Cluster of differentiation 40, tumor necrosis factor receptor superfamily member 5, soluble
sCD244	Natural killer cell receptor 2B4, soluble
sCDCP1	CUB domain-containing protein 1, soluble
sCX3CL1	C-X3-C motif chemokine ligand 1, fractalkine, soluble
sDNER	Delta and Notch-like epidermal growth factor-related receptor, soluble
sFlt3L	Fms-related tyrosine kinase 3 ligand, soluble
sHGF	Hepatocyte growth factor
sIL2RB	Interleukin-2 receptor subunit beta, soluble
sIL10RA	Interleukin-10 receptor subunit alpha, soluble
sIL10RB	Interleukin-10 receptor subunit beta, soluble
sIL15RA	Interleukin-15 receptor subunit alpha, soluble
sIL18R1	Interleukin-18 receptor 1, soluble
sIL20RA	Interleukin-20 receptor subunit alpha, soluble
sIL22RA1	Interleukin-22 receptor subunit alpha-1, soluble
SIRT2	Sirtuin 2
sLIFR	Leukemia inhibitory factor receptor, soluble
sOPG	Osteoprotegerin, soluble
sPDL1	Programmed cell death 1 ligand 1, soluble
sSCF	Stem cell factor, soluble
sSLAMF1	Signaling lymphocytic activation molecule, soluble
ST1A1	Sulfotransferase 1A1

STAMBP	STAM-binding protein
sTGFA	Transforming growth factor alpha, soluble
sTNFB	TNF-beta, soluble
sTNFRSF9	Tumor necrosis factor receptor superfamily member 9, soluble
sTNFSF14	Tumor necrosis factor ligand superfamily member 14, soluble
sTRAIL	TNF-related apoptosis-inducing ligand, soluble
sTRANCE	TNF-related activation-induced cytokine, soluble
sTWEAK	Tumor necrosis factor ligand superfamily, member 12
TSLP	Thymic stromal lymphopoietin
TNF	Tumor necrosis factor
uPA	Urokinase-type plasminogen activator
VEGFA	Vascular endothelial growth factor A

Table S2 Serum levels of studied proteins in a) healthy controls *vs* SLE patients, b) SLE patients with organ damage (SDI≥1) *vs* those without organ damage (SDI=0), c) patients with biopsy-proven lupus nephritis (LN) *vs* patients without lupus nephritis (no LN).

a) Healthy controls *vs* SLE

Analyte	*Mean Linear ddCq (95 % CI)*		*FC*	*P*	P_{corr}
	Healthy controls	*SLE*			
SIRT2	8.31 (6.49-10.1)	19.8 (15.5-24.0)	2.33	6.5×10^{-6}	5.1×10^{-4}
IL18	183 (155-212)	287 (257-316)	1.67	1.6×10^{-5}	6.2×10^{-4}
CASP8	2.04 (1.88-2.20)	2.99 (2.68-3.30)	1.37	2.5×10^{-5}	6.3×10^{-4}
sCD40	527 (466-588)	735 (639-831)	1.29	3.2×10^{-5}	6.3×10^{-4}
sSLAMF1	5.10 (4.0-6.19)	6.52 (6.0-7.05)	1.39	9.0×10^{-5}	1.1×10^{-3}
sTNFRSF9	87.8 (75.2-100)	141 (123-159)	1.54	1.1×10^{-4}	1.1×10^{-3}
ST1A1	3.36 (2.04-4.69)	8.04 (6.66-9.43)	2.41	1.3×10^{-4}	1.1×10^{-3}
STAMBP	12.1 (10.1-14.1)	18.9 (16.1-21.8)	1.42	1.5×10^{-4}	1.1×10^{-3}
CCL19	804 (394-1215)	1646 (1326-1966)	2.04	1.5×10^{-4}	1.1×10^{-3}
IL10	8.33 (6.94-9.72)	17.8 (10.4-25.1)	1.38	3.7×10^{-4}	2.6×10^{-3}
CCL4	77.7 (63.8-91.6)	123 (109-138)	1.46	4.2×10^{-4}	2.7×10^{-3}
IL12B	17.3 (13.3-21.4)	29.3 (25.1-33.5)	1.96	5.7×10^{-4}	3.4×10^{-3}
IL6	4.18 (3.28-5.09)	26.9 (-0.62-54.4)	1.67	7.0×10^{-4}	3.9×10^{-3}
CCL3	9.58 (4.47-14.7)	35.8 (-11.2-82.9)	1.51	7.6×10^{-4}	4.0×10^{-3}
CXCL11	187 (142-232)	343 (279-408)	1.58	1.1×10^{-3}	5.4×10^{-3}
sPDL1	3.43 (3.19-3.67)	4.24 (3.91-4.57)	1.28	1.2×10^{-3}	5.4×10^{-3}
sIL18R1	106 (90.1-122)	139 (126-152)	1.28	1.4×10^{-3}	6.0×10^{-3}
sCX3CL1	86.9 (74.8-99.0)	131 (108-153)	1.39	2.2×10^{-3}	9.0×10^{-3}
sDNER	170 (161-179)	150 (143-157)	0.91	2.6×10^{-3}	1.0×10^{-2}
sIL15RA	1.90 (1.68-2.13)	2.43 (2.19-2.66)	1.23	2.9×10^{-3}	1.1×10^{-2}
CSF1	241 (223-259)	281 (270-293)	1.11	3.3×10^{-3}	1.2×10^{-2}
sLIFR	8.35 (7.84-8.85)	10.3 (8.94-11.6)	1.04	3.4×10^{-3}	1.2×10^{-2}
IL8	236 (199-273)	383 (281-485)	1.27	3.9×10^{-3}	1.2×10^{-2}
CCL2	2133 (1801-2465)	3054 (2660-3449)	1.26	3.9×10^{-3}	1.2×10^{-2}
FGF23	2.77 (2.63-2.91)	4.32 (2.95-5.70)	1.01	5.8×10^{-3}	1.8×10^{-2}
LAP.TGFB1	131 (117-146)	158 (148-167)	1.28	6.6×10^{-3}	1.9×10^{-2}
sTRAIL	517 (470-565)	602 (568-636)	1.20	1.1×10^{-2}	3.1×10^{-2}
MMP10	98.5 (81.5-116)	156 (128-184)	1.34	1.4×10^{-2}	3.7×10^{-2}
CCL7	5.70 (4.57-6.83)	12.5 (7.31-17.7)	1.38	1.6×10^{-2}	4.1×10^{-2}
IL7	20.3 (17.3-23.3)	16.9 (15.4-18.3)	0.82	2.3×10^{-2}	5.5×10^{-2}
sTRANCE	23.8 (19.6-28.1)	34.2 (29.4-38-9)	1.24	2.3×10^{-2}	5.5×10^{-2}
CXCL10	312 (226-397)	575 (369-782)	1.53	2.6×10^{-2}	6.0×10^{-2}
CCL28	2.71 (2.37-3.05)	2.35 (2.22-2.49)	0.88	3.1×10^{-2}	7.2×10^{-2}
sCDCP1	9.34 (7.01-11.7)	10.5 (9.20-11.8)	1.15	4.0×10^{-2}	8.8×10^{-2}
sTNFSF14	18.1 (13.9-22.3)	24.6 (21.6-27.7)	1.35	4.0×10^{-2}	8.8×10^{-2}
CXCL5	4528 (3582-5474)	3589 (3179-3999)	0.84	4.4×10^{-2}	9.3×10^{-2}
IFNG	1.99 (1.71-2.26)	3.98 (1.94-6.03)	1.18	4.7×10^{-2}	9.7×10^{-2}
NT3	3.74 (3.24-4.23)	3.45 (2.97-3.92)	0.84	5.2×10^{-2}	0.10
bNGF	2.51 (2.24-2.77)	2.83 (2.64-3.01)	1.12	5.8×10^{-2}	0.11

FGF21	67.9 (25.7-110)	168 (55.7-281)	1.59	6.8×10^{-2}	0.13
FGF19	308 (214-402)	240 (196-285)	0.67	6.8×10^{-2}	0.13
CXCL9	157 (87.9-226)	190 (137-242)	1.26	0.10	0.18
sSCF	236 (205-266)	261 (244-278)	1.20	0.11	0.19
CCL13	16.0 (12.7-19.2)	13.3 (11.9-14.6)	0.82	0.11	0.19
IL17C	6.70 (2.57-10.8)	8.91 (5.74-12.1)	1.18	0.11	0.19
sIL22RA1	2.39 (2.34-2.45)	3.38 (1.91-4.86)	1.00	0.12	0.19
CXCL6	315 (255-376)	384 (334-434)	1.12	0.17	0.27
CXCL1	468 (393-543)	548 (500-597)	1.01	0.19	0.31
CCL11	282 (242-322)	313 (292-334)	1.03	0.21	0.33
sADA	40.0 (33.4-46.6)	34.8 (32.3-37.3)	0.97	0.22	0.34
sHGF	185 (166-205)	181 (164-199)	0.91	0.23	0.35
VEGFA	3437 (2936-3938)	3990 (3603-4377)	1.07	0.27	0.39
sCD6	15.8 (12.7-18.8)	14.1 (12.7-15.6)	0.85	0.27	0.39
sCD5	18.6 (16.0-21.2)	20.8 (18.9-22.6)	1.07	0.28	0.40
CCL20	62.3 (42.7-82.0)	145 (72.7-216)	1.34	0.29	0.41
CCL8	808 (651-964)	1043 (860-1226)	1.12	0.32	0.44
uPA	1174 (1094-1254)	1221 (1168-1274)	1.04	0.33	0.44
4E.BP1	39.2 (35.3-53.2)	60.1 (39.4-80.8)	1.19	0.33	0.44
GDNF	4.90 (4.22-5.59)	5.07 (4.75-5.38)	1.07	0.37	0.48
FGF5	2.64 (2.25-3.02)	2.51 (2.27-2.75)	0.98	0.38	0.48
BDNF	811 (247-1375)	816 (513-1119)	0.44	0.44	0.55
IL24	1.92 (1.67-2.17)	2.13 (1.82-2.45)	1.00	0.45	0.55
sTNFB	13.6 (12.0-15.1)	14.7 (13.5-16.0)	1.03	0.47	0.57
sOPG	1103 (995-1211)	1186 (1100-1272)	1.03	0.53	0.63
sIL10RA	2.25 (2.05-2.46)	3.09 (1.89-4.29)	1.00	0.54	0.64
CCL25	84.9 (71.2-98.6)	94.5 (84.0-105)	1.17	0.55	0.64
CCL23	757.0 (636.6-877.4)	739.7 (661.6-817.8)	1.007	0.65	0.74
EN.RAGE	20.4 (16.9-23.8)	27.2 (21.3-33.1)	1.05	0.66	0.75
OSM	42.6 (34.1-51.1)	51.5 (40.5-62.4)	1.15	0.73	0.81
sTGFA	9.31 (7.47-11.1)	11.5 (8.53-14.6)	1.05	0.74	0.81
sIL10RB	85.2 (70.9-99.6)	80.1 (73.6-86.5)	0.92	0.78	0.85
sTWEAK	661 (600-722)	652 (618-686)	1.00	0.79	0.85
IL17A	1.57 (1.33-1.81)	2.14 (1.18-3.09)	1.04	0.83	0.87
MMP1	10.2 (7.55-12.9)	10.1 (8.66-11.5)	0.99	0.90	0.93
sCD244	74.1 (66.0-82.3)	71.7 (68.4-75.0)	0.99	0.94	0.96
sFlt3L	513 (465-561)	552 (489-616)	0.99	0.94	0.96
CST5	105 (88.6-122)	108 (98.0-118)	1.06	0.98	0.98

b) SDI=0 *vs* SDI≥1

Analyte	*Mean Linear ddCq (95 % CI)*		*FC*	*P*	P_{corr}
	SDI=0	*SDI≥1*			
IL8	286 (215-358)	459 (286-632)	1.32	2.7×10^{-5}	2.1×10^{-3}
CCL2	2485 (2011-2960)	3502 (2924-4080)	1.50	4.3×10^{-4}	1.6×10^{-2}
IL6	8.28 (3.95-12.6)	41.5 (-7.95-91.0)	1.98	6.5×10^{-4}	1.6×10^{-2}
CCL11	273 (247-299)	344 (315-373)	1.30	8.0×10^{-4}	1.6×10^{-2}
FGF21	56.1 (28.8-83.4)	257 (57.1-456)	2.43	1.0×10^{-3}	1.6×10^{-2}
MMP10	112 (93.4-131)	190 (144-237)	1.24	2.4×10^{-3}	3.1×10^{-2}

IL18	255 (208-302)	311 (274-349)	1.19	3.7×10^{-3}	4.1×10^{-2}
CCL3	9.70 (8.24-11.2)	56.4 (-28.7-142)	1.32	4.8×10^{-3}	4.4×10^{-2}
FGF5	2.25 (2.13-2.37)	2.72 (2.31-3.12)	1.08	5.1×10^{-3}	4.4×10^{-2}
FGF23	3.75 (2.51-5.00)	4.77 (2.47-7.06)	1.15	5.7×10^{-3}	4.4×10^{-2}
IL7	18.6 (16.4-20.8)	15.5 (13.5-17.4)	0.80	1.5×10^{-2}	9.8×10^{-2}
IL17C	9.83 (2.74-16.9)	8.19 (6.49-9.89)	1.26	1.5×10^{-2}	9.8×10^{-2}
sCDCP1	10.0 (7.57-12.4)	10.9 (9.47-12.4)	1.18	2.1×10^{-2}	0.12
VEGFA	3540 (3034-4046)	4343 (3782-4904)	1.25	3.2×10^{-2}	0.18
NT3	3.86 (2.82-4.91)	3.12 (2.87-3.37)	0.92	3.7×10^{-2}	0.19
CCL7	8.91 (5.39-12.4)	15.3 (6.37-24.3)	1.29	4.0×10^{-2}	0.19
IL10	13.0 (9.86-16.1)	21.5 (8.46-34.6)	1.20	6.7×10^{-2}	0.30
sIL15RA	2.27 (1.93-2.62)	2.54 (2.21-2.87)	1.18	7.2×10^{-2}	0.30
4E.BP1	52.2 (24.9-79.4)	66.4 (35.4-97.4)	1.55	7.2×10^{-2}	0.30
CXCL5	3845 (3347-4343)	3388 (2758-4018)	0.80	7.9×10^{-2}	0.31
sTNFB	15.6 (13.9-17.3)	14.0 (12.2-15.9)	0.88	9.5×10^{-2}	0.35
CXCL9	157 (82.7-230.9)	215 (139-291)	1.20	0.10	0.35
sTNFRSF9	122 (106-137)	156 (127-186)	1.25	0.10	0.35
BDNF	1043 (475-1611)	637 (317-958)	0.05	0.11	0.35
uPA	1180 (1112-1248)	1254 (1174-1334)	1.09	0.13	0.39
CSF1	272 (255-290)	288 (272-304)	1.08	0.13	0.39
OSM	54.3 (30.6-78.0)	49.3 (41.8-56.7)	1.25	0.14	0.39
CCL25	83.9 (71.0-96.8)	103 (87.1-119)	1.11	0.15	0.39
sIL10RB	72.8 (65.6-80.0)	85.8 (75.9-95.8)	1.04	0.15	0.39
CCL20	105 (49.0-161)	176 (53.3-298)	1.69	0.15	0.39
sIL22RA1	2.89 (4.43-3.34)	3.77 (1.11-6.43)	1.00	0.15	0.39
GDNF	4.81 (4.42-5.21)	5.26 (4.79-5.74)	1.06	0.17	0.41
sCD40	660 (602-717)	794 (627-961)	1.05	0.18	0.42
sSLAMF1	6.33 (5.42-7.23)	6.68 (6.03-7.32)	1.08	0.18	0.42
sTGFA	13.1 (6.36-19.9)	10.3 (8.86-11.7)	1.22	0.21	0.45
bNGF	2.74 (2.44-3.04)	2.89 (2.65-3.13)	1.15	0.22	0.45
LAP.TGFB1	151 (138-165)	162 (150-175)	1.07	0.22	0.45
sHGF	184 (149-220)	179 (163-195)	1.11	0.22	0.45
sTWEAK	679 (633-726)	631 (582-681)	0.95	0.23	0.46
sIL18R1	139 (112-165)	139 (127-151)	1.08	0.24	0.46
CXCL6	364 (279-450)	399 (337-461)	1.14	0.25	0.47
CCL8	1074 (821-1327)	1018 (749-1287)	0.82	0.28	0.51
CCL13	12.3 (10.3-14.2)	14.1 (12.2-16.0)	1.29	0.29	0.51
CASP8	2.87 (2.40-3.34)	3.08 (2.66-3.50)	1.12	0.30	0.51
CCL19	1562 (1030-2095)	1712 (1304-2120)	1.19	0.30	0.51
sIL10RA	2.57 (2.24-2.91)	3.50 (1.34-5.65)	1.00	0.31	0.53
sDNER	153 (144-163)	147 (137-157)	0.99	0.33	0.55
sPDL1	4.10 (3.60-4.60)	4.36 (3.90-4.81)	1.06	0.36	0.58
sFlt3L	491 (439-544)	600 (496-705)	1.007	0.38	0.59
sLIFR	11.0 (7.99-14.0)	9.74 (9.01-10.5)	1.05	0.38	0.59
sTRAIL	623 (558-689)	585 (551-618)	0.98	0.43	0.66
CCL23	721 (604-838)	755 (645-864)	1.08	0.44	0.66
sCD6	14.5 (12.4-16.6)	13.9 (11.9-15.9)	0.92	0.46	0.67
CST5	101 (89.6-112)	114 (97.7-129)	0.92	0.46	0.67

sSCF	266 (241-290)	258 (234-281)	0.98	0.48	0.68
sCD5	20.1 (17.6-22.5)	21.3 (18.6-24.1)	1.17	0.50	0.69
sOPG	1168 (1034-1301)	1199 (1083-1315)	1.01	0.51	0.69
CCL4	118 (96.0-140)	128 (108-149)	0.98	0.51	0.69
EN.RAGE	30.4 (19.0-41.8)	24.7 (18.7-30.6)	0.91	0.53	0.69
CCL28	2.42 (2.18-2.66)	2.30 (2.14-2.46)	0.98	0.56	0.73
MMP1	10.3 (8.19-12.3)	9.93 (7.94-11.9)	0.97	0.61	0.78
sADA	35.6 (31.7-39.5)	34.1 (30.7-37.5)	0.96	0.68	0.81
sTNFSF14	23.8 (19.0-28.6)	25.3 (21.2-29.4)	1.06	0.68	0.81
IFNG	5.29 (0.82-9.76)	2.95 (1.70-4.20)	1.11	0.68	0.81
FGF19	218 (178-258)	258 (185-332)	0.91	0.69	0.81
CXCL10	491 (322-660)	642 (291-993)	1.29	0.69	0.81
CXCL11	342 (254-429)	345 (249-441)	0.85	0.70	0.82
IL24	1.93 (1.67-2.18)	2.30 (1.77-2.82)	1.00	0.71	0.82
AXIN1	4.12 (3.49-4.74)	4.57 (3.76-5.38)	0.98	0.77	0.87
sTRANCE	35.5 (27.3-43.7)	33.1 (27.3-38.8)	0.90	0.81	0.90
sCX3CL1	123 (90.3-157)	136 (105-168)	1.02	0.82	0.90
SIRT2	19.3 (13.3-25.2)	20.1 (14.0-26.3)	0.98	0.85	0.92
STAMBP	18.3 (14.6-22.0)	19.4 (15.1-23.7)	0.85	0.88	0.94
IL12B	30.8 (23.3-38.2)	28.2 (23.3-33.1)	1.08	0.89	0.94
CXCL1	554 (471-637)	544 (484-604)	0.996	0.93	0.96
IL17A	1.69 (1.39-2.00)	2.49 (0.77-4.21)	0.98	0.96	0.98
sCD244	72.2 (67.9-76.5)	71.3 (66.3-76.4)	1.00	0.97	0.98
ST1A1	8.05 (5.97-10.1)	8.04 (6.10-9.98)	0.86	0.99	0.99

c) no LN *vs* LN

	Mean Linear ddCq (95 % CI)		*FC*	*P*	*P_{corr}*
	no LN	*LN*			
sCD40	645 (595-695)	894 (643-1146)	1.17	2.9×10^{-4}	1.2×10^{-2}
GDNF	4.66 (4.37-4.96)	5.78 (5.14-6.43)	1.21	2.9×10^{-4}	1.2×10^{-2}
sCX3CL1	103 (93.2-112)	180 (123-238)	1.33	1.6×10^{-3}	4.1×10^{-2}
sIL10RB	73.4 (66.2-80.5)	92.0 (80.1-104)	1.31	2.6×10^{-3}	4.1×10^{-2}
CSF1	266 (254-278)	309 (287-331)	1.14	2.6×10^{-3}	4.1×10^{-2}
sPDL1	3.86 (3.58-4.15)	4.92 (4.19-5.65)	1.22	3.5×10^{-3}	4.5×10^{-2}
CASP8	2.80 (2.39-3.21)	3.32 (2.87-3.77)	1.23	5.1×10^{-3}	5.7×10^{-2}
bNGF	2.66 (2.47-2.84)	3.13 (2.74-3.52)	1.18	2.5×10^{-2}	0.21
FGF19	210 (165-256)	294 (201-387)	1.22	2.8×10^{-2}	0.21
sIL15RA	2.17 (2.03-2.32)	2.87 (2.28-3.46)	1.16	2.8×10^{-2}	0.21
IL24	2.12 (1.67-2.57)	2.15 (1.78-2.53)	1.00	3.0×10^{-2}	0.21
LAP.TGFB1	150 (139-161)	171 (154-187)	1.13	3.7×10^{-2}	0.21
AXIN1	3.99 (3.37-4.61)	5.04 (4.10-5.99)	1.31	3.8×10^{-2}	0.21
sIL18R	129 (119-139)	156 (124-188)	1.14	3.8×10^{-2}	0.21
MMP10	137 (111-164)	189 (124-253)	1.31	4.8×10^{-2}	0.25
CCL13	14.4 (12.5-16.3)	11.3 (9.66-13.0)	0.70	5.1×10^{-2}	0.25
sCD6	15.0 (13.2-16.8)	12.7 (10.3-15.0)	0.80	6.4×10^{-2}	0.29
CCL8	924 (743-1105)	1254 (853-1655)	1.39	6.6×10^{-2}	0.29
IL8	326 (269-383)	484 (213-756)	1.09	7.1×10^{-2}	0.29

STAMBP	18.6 (14.3-22.9)	19.5 (16.9-22.1)	1.26	7.6×10^{-2}	0.29
NT3	3.13 (2.91-3.35)	4.01 (2.73-5.30)	1.14	7.9×10^{-2}	0.29
sSCF	253 (233-273)	276 (245-308)	1.13	0.10	0.35
sLIFR	9.17 (8.85-9.49)	12.3 (8.55-16.0)	1.04	0.10	0.35
CST5	102 (90.3-113)	120 (99.6-139)	1.30	0.11	0.36
SIRT2	19.3 (13.1-25.5)	20.5 (15.8-25.2)	1.29	0.12	0.37
VEGFA	3754 (3317-4191)	4409 (3650-5168)	1.27	0.13	0.39
CXCL1	528 (470-586)	585 (494-675)	1.15	0.15	0.43
IL10	13.0 (10.5-15.6)	26.2 (5.77-46.6)	1.08	0.17	0.45
4E.BP1	64.2 (32.3-96.0)	53.0 (39.5-66.5)	1.34	0.17	0.45
IL7	16.2 (14.3-18.1)	18.0 (15.6-20.4)	1.25	0.18	0.45
CXCL11	305 (236-374)	411 (277-546)	1.15	0.18	0.45
BDNF	707 (374-1041)	1009 (384-1634)	3.97	0.19	0.45
IL18	268 (238-298)	320 (257-383)	1.09	0.20	0.46
sTRANCE	31.5 (26.5-36.4)	38.9 (29.0-48.9)	1.13	0.20	0.46
sTNFSF14	23.3 (19.6-27.0)	27.0 (21.4-32.6)	1.26	0.22	0.49
sTRAIL	592 (547-638)	618 (566-670)	1.26	0.27	0.57
CCL25	88.5 (77.2-99.9)	105 (83.6-127)	1.13	0.28	0.57
sSLAMF1	6.22 (5.67-6.78)	7.06 (5.95-8.16)	0.99	0.28	0.57
CCL2	2866 (2455-3277)	3389 (2544-4234)	1.10	0.30	0.59
sTNFRSF9	135 (115-156)	151 (116-187)	1.12	0.30	0.59
ST1A1	7.56 (5.83-9.29)	8.90 (6.48-11.3)	1.29	0.32	0.61
sTNFB	15.1 (13.5-16.7)	14.0 (11.8-16.2)	0.91	0.33	0.61
IL6	14.3 (6.89-21.7)	49.3 (-28.5-127)	1.09	0.34	0.61
sADA	33.0 (30.7-35.4)	37.8 (32.1-43.5)	1.11	0.36	0.62
sCD5	20.3 (18.1-22.5)	21.6 (18.3-25.0)	1.14	0.37	0.62
sHGF	173 (156-191)	196 (156-235)	1.08	0.37	0.62
sOPG	1132 (1055-1210)	1281 (1083-1479)	1.01	0.39	0.62
CCL3	48.0 (-26.3-122)	14.2 (10.2-18.2)	0.97	0.39	0.62
CXCL9	171 (128-214)	222 (92.1-353)	0.80	0.40	0.62
CXCL10	470 (340-600)	763 (221-1305)	1.06	0.40	0.62
sIL10RA	3.31 (1.44-5.19)	2.69 (2.27-3.11)	1.00	0.40	0.62
MMP1	9.33 (7.81-10.9)	11.4 (8.50-14.3)	1.29	0.42	0.63
CCL7	10.5 (5.44-15.5)	16.1 (4.33-28.0)	1.03	0.44	0.64
CCL11	310 (282-339)	317 (286-349)	1.09	0.44	0.64
sIL22RA1	3.79 (1.47-6.10)	2.66 (2.20-3.12)	1.00	0.47	0.67
CXCL5	3698 (3193-4203)	3395 (2655-4136)	1.09	0.50	0.70
IFNG	2.68 (1.87-3.48)	6.30 (0.67-11.9)	0.98	0.51	0.70
sCDCP1	10.1 (8.65-11.6)	11.2 (8.55-13.9)	1.02	0.52	0.70
EN.RAGE	25.1 (17.7-32.6)	30.8 (20.6-41.1)	0.90	0.53	0.70
OSM	46.4 (37.5-55.4)	60.5 (33.7-87.2)	1.05	0.56	0.72
FGF21	149 (3.51-294)	203 (14.4-392)	0.83	0.57	0.72
sTGFA	10.0 (8.11-12.0)	14.2 (6.34-22.1)	0.93	0.60	0.75
sFlt3L	573 (483-663)	516 (439-594)	0.94	0.62	0.75
CCL23	706 (624-788)	799 (632-966)	1.09	0.63	0.75
CCL20	121 (64.7-178)	186 (6.44-365)	0.77	0.63	0.75
sCD244	71.6 (67.4-75.9)	71.8 (66.0-77.6)	1.07	0.65	0.75
IL12B	30.5 (24.5-36.4)	27.3 (22.1-32.5)	0.83	0.66	0.75

IL17C	9.53 (4.66-14.4)	7.81 (5.62-10.0)	1.08	0.66	0.75
FGF5	2.50 (2.16-2.85)	2.53 (2.27-2.78)	1.00	0.67	0.75
CCL28	2.30 (2.16-2.44)	2.45 (2.15-2.74)	1.08	0.67	0.75
CCL19	1708 (1276-2140)	1535 (1056-2014)	0.99	0.70	0.76
sDNER	150 (142-159)	150 (137-162)	0.99	0.75	0.81
CCL4	122 (104-140)	127 (101-153)	0.97	0.81	0.87
CXCL6	387 (321-453)	378 (297-459)	1.01	0.89	0.93
IL17A	1.65 (1.43-1.87)	3.0 (0.29-5.71)	0.97	0.94	0.98
FGF23	3.48 (2.99-3.96)	5.82 (2.01-9.64)	0.98	0.97	0.99
sTWEAK	657 (620-694)	644 (573-716)	1.03	0.97	0.99
uPA	1226 (1161-1291)	1213 (1114-1312)	0.99	0.99	0.99

P_{corr} value corrected for multiple comparisons (Benjamini-Hochberg correction)

FC (Fold Change) between group medians of linear ddCq. Upregulated proteins have values above 1, downregulated proteins have values between 0 to 1.

Table S3 Correlation analysis for a) serum levels of studied proteins with the disease activity as assessed by SLEDAI and the cumulative dose of glucocorticoids; and b) IFN serum protein "signature" in SLE patients with the disease activity as assessed by SLEDAI.

**a)**	**SLEDAI**		**Cumulative dose of glucocorticoids**	
Protein	Spearman r	*P* value	Spearman r	*P* value
4EBP1	0.186	0.111	0.109	0.352
AXIN1	-0.060	0.611	-0.026	0.826
BDNF	0.013	0.914	-0.238	0.040
bNGF	0.282	0.014	0.198	0.089
CASP8	0.315	0.006	0.105	0.368
CCL11	0.261	0.024	0.371	0.001
CCL13	-0.208	0.074	0.057	0.626
CCL19	0.058	0.620	-0.065	0.579
CCL2	0.276	0.017	0.249	0.031
CCL20	0.209	0.072	0.064	0.587
CCL23	0.236	0.042	-0.203	0.080
CCL25	-0.028	0.814	0.300	0.009
CCL28	0.162	0.165	0.039	0.739
CCL3	0.090	0.443	0.101	0.391
CCL4	0.032	0.787	0.022	0.849
CCL7	0.393	0.001	0.164	0.160
CCL8	0.052	0.658	-0.171	0.141
CSF1	0.311	0.007	0.209	0.072
CST5	0.136	0.244	0.035	0.765
CXCL1	0.155	0.186	0.234	0.043
CXCL10	0.169	0.147	-0.048	0.684
CXCL11	0.245	0.034	-0.116	0.321
CXCL5	-0.107	0.359	-0.017	0.883
CXCL6	-0.126	0.280	0.198	0.089
CXCL9	0.053	0.649	0.027	0.821
ENRAGE	0.064	0.586	-0.189	0.105
FGF19	0.104	0.373	0.068	0.563
FGF21	0.345	0.002	0.042	0.718
FGF23	0.307	0.007	0.199	0.086
FGF5	0.205	0.077	0.157	0.179
GDNF	0.405	0.001	0.238	0.040
IFNG	0.094	0.422	0.030	0.796
IL10	0.286	0.013	0.119	0.310
IL12B	-0.064	0.583	-0.016	0.895
IL17A	0.217	0.062	0.169	0.147
IL17C	0.319	0.005	0.251	0.030
IL18	0.318	0.006	0.220	0.058
IL24	0.213	0.067	-0.027	0.816
IL6	0.345	0.003	0.098	0.405
IL7	0.123	0.294	-0.036	0.762
IL8	0.416	0.001	0.326	0.004

LAP.TGFB1	0.040	0.731	0.218	0.060
MMP1	0.023	0.848	-0.139	0.234
MMP10	0.355	0.002	0.240	0.038
NT3	0.105	0.372	-0.156	0.182
OSM	-0.089	0.450	0.113	0.333
sADA	0.109	0.351	-0.241	0.037
sCD244	-0.238	0.040	0.067	0.569
sCD40	0.220	0.058	0.090	0.443
sCD5	0.134	0.253	0.072	0.539
sCD6	-0.287	0.013	0.035	0.763
sCDCP1	0.270	0.019	0.238	0.040
sCX3CL1	0.403	0.001	0.106	0.363
sDNER	-0.217	0.062	0.143	0.222
sFlt3L	-0.002	0.983	0.057	0.626
sHGF	-0.062	0.600	0.054	0.646
sIL10RA	-0.018	0.879	-0.068	0.561
sIL10RB	0.251	0.030	0.149	0.203
sIL15RA	0.383	0.001	0.207	0.075
sIL18R1	0.258	0.025	0.237	0.040
sIL22RA1	0.072	0.541	-0.102	0.386
SIRT2	-0.063	0.592	-0.005	0.963
sLIFR	0.306	0.008	0.129	0.270
sOPG	0.212	0.069	0.005	0.968
sPDL1	0.240	0.038	0.028	0.812
sSCF	-0.166	0.154	0.253	0.028
sSLAMF1	0.160	0.170	-0.018	0.876
ST1A1	-0.203	0.081	0.053	0.650
STAMPB	-0.043	0.711	0.063	0.589
sTGFA	-0.074	0.528	0.280	0.015
sTNFB	-0.223	0.054	-0.210	0.071
sTNFRSF9	0.096	0.414	0.002	0.988
sTNFSF14	-0.102	0.384	0.107	0.362
sTRAIL	0.027	0.820	-0.065	0.579
sTRANCE	0.095	0.418	0.003	0.978
sTWEAK	-0.251	0.030	-0.037	0.751
uPA	0.000	0.997	0.127	0.277
VEGFA	0.373	0.001	0.155	0.183

b)

IFN signature	0.252	0.029

Figure S1 Distribution of damage according to the involved organ/system within Czech patients with organ damage and comparison with reported European and Hopkins Lupus cohorts [1,2].

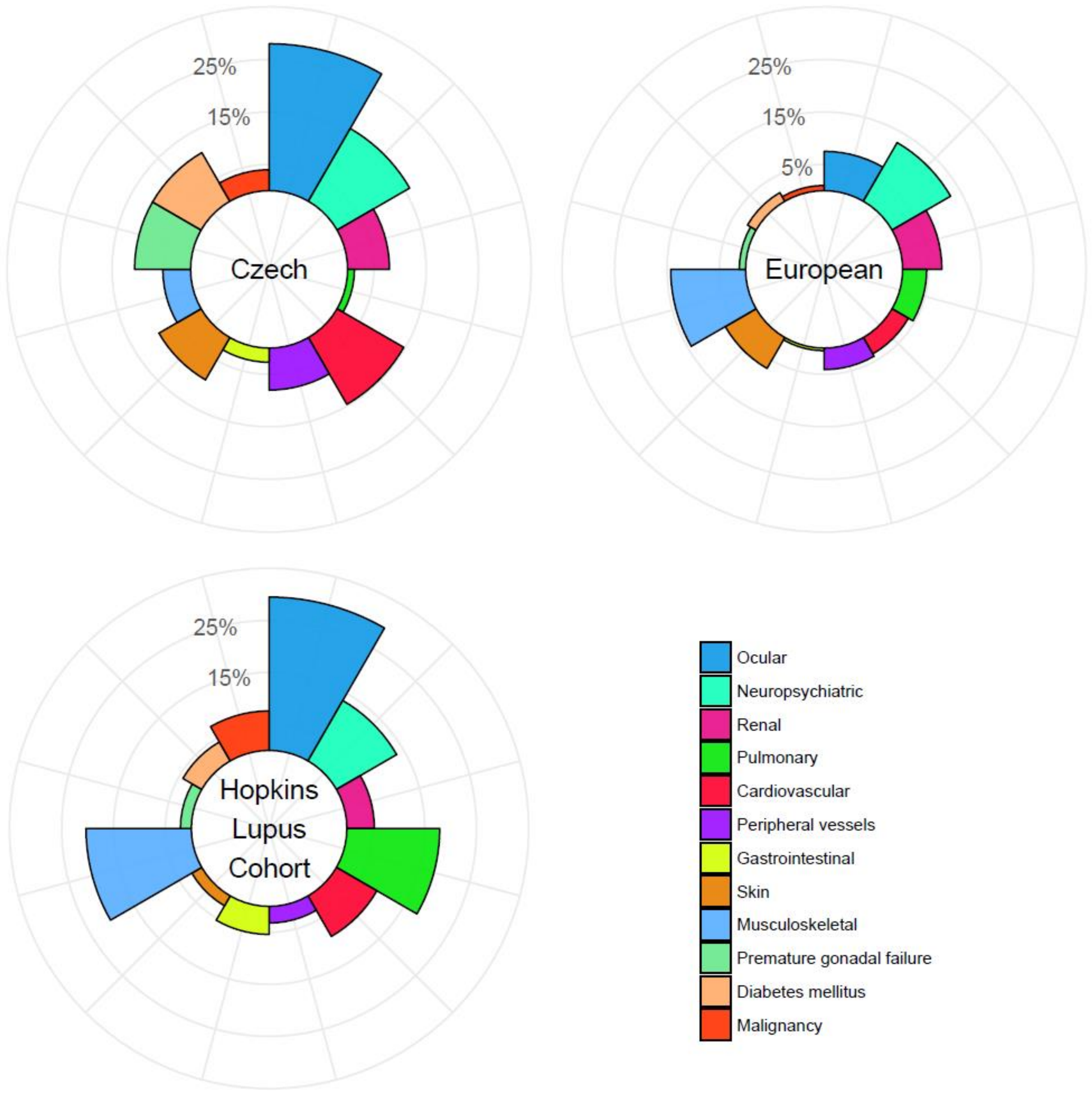

Table S4 Distribution of damage according to the involved organ/system within systemic lupus erythematosus (SLE) patients with organ damage. Organ damage was assessed by SDI damage index (Systemic Lupus International Collaborating Clinics/American College of Rheumatology Damage Index).

SDI domains	*Patients* N *(%)*
Ocular	21 (28.0 %)
Neuropsychiatric	12 (16.0 %)
Renal	6 (8.0 %)
Pulmonary	1 (1.3 %)
Cardiovascular	11 (14.7 %)
Peripheral vessels	6 (8.0 %)
Gastrointestinal	2 (2.7 %)
Skin	7 (9.3 %)
Musculoskeletal	4 (5.3 %)
Premature gonadal failure	8 (10.7 %)
Diabetes mellitus	8 (10.7 %)
Malignancy	3 (4.0 %)

Figure S2 Comparison of serum levels of a) CCL11 between subgroups of SLE patients with/without organ damage and healthy controls and b) GDNF between subgroups of SLE patients with/without LN and healthy control subjects

a)

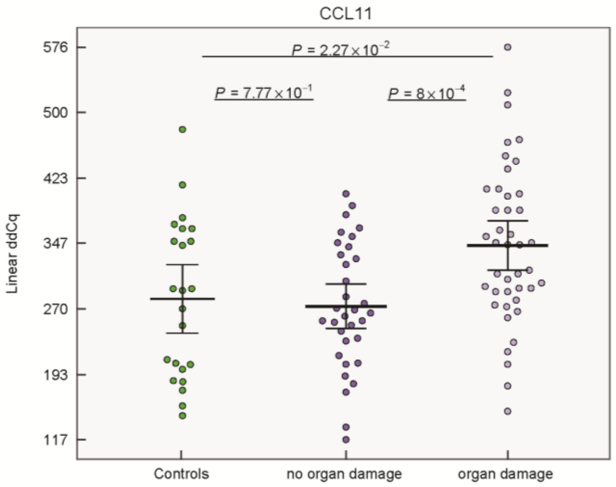

b)

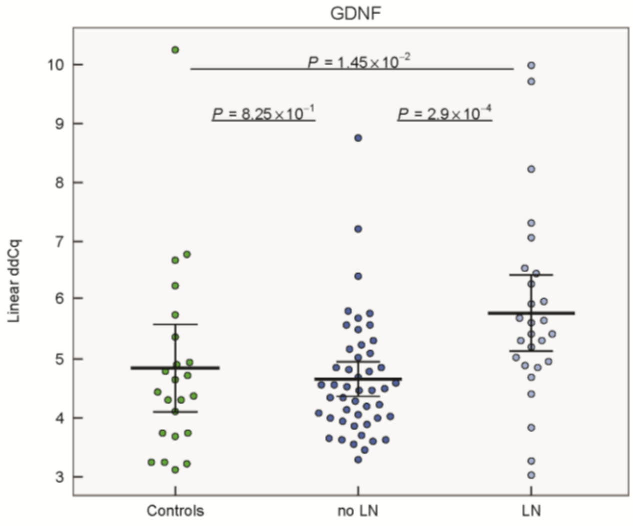

Figure S3 ROC analysis of serum proteins for a) organ damage *vs* no organ damage, b) active LN *vs* no LN, and c) active LN *vs* inactive LN.

a)

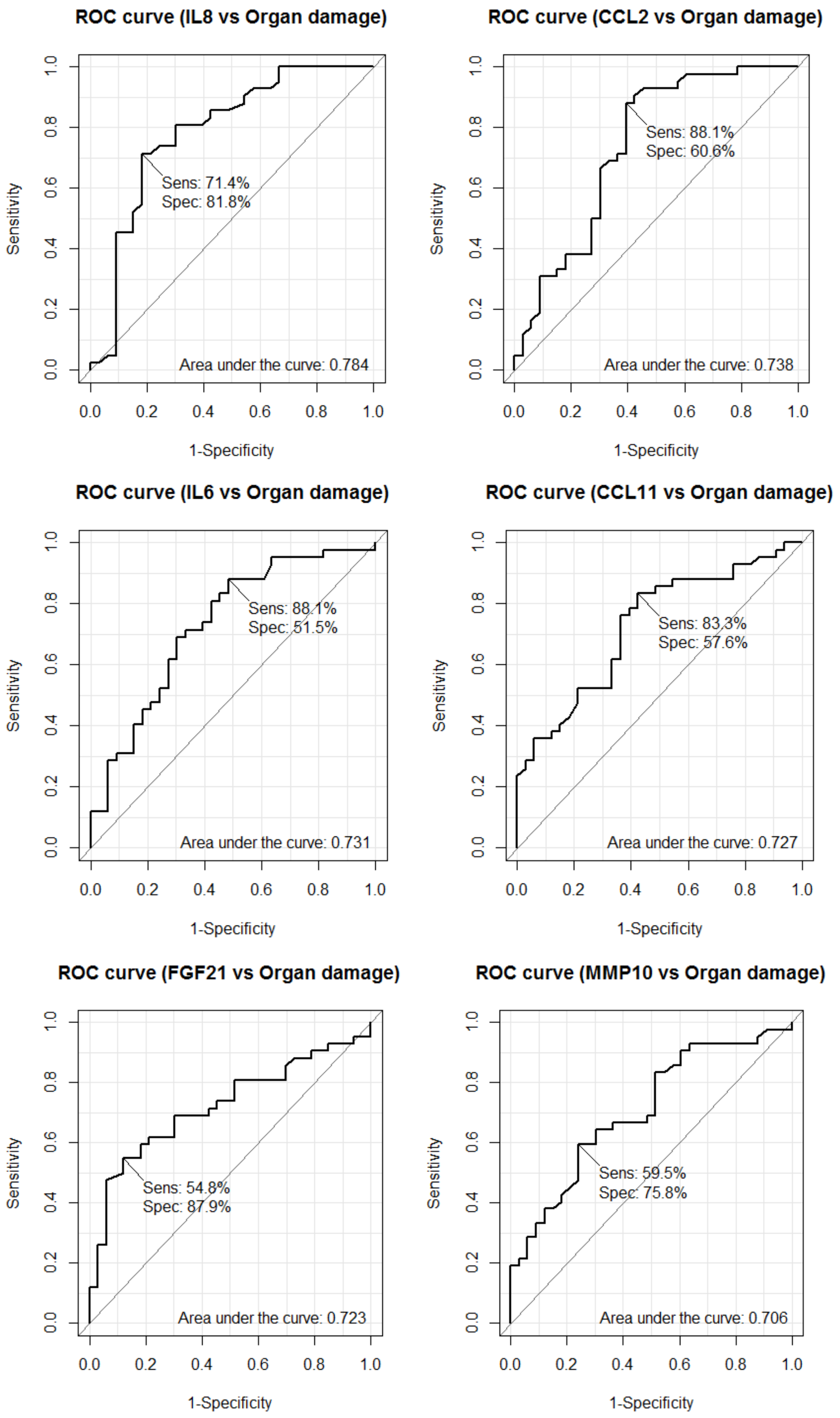

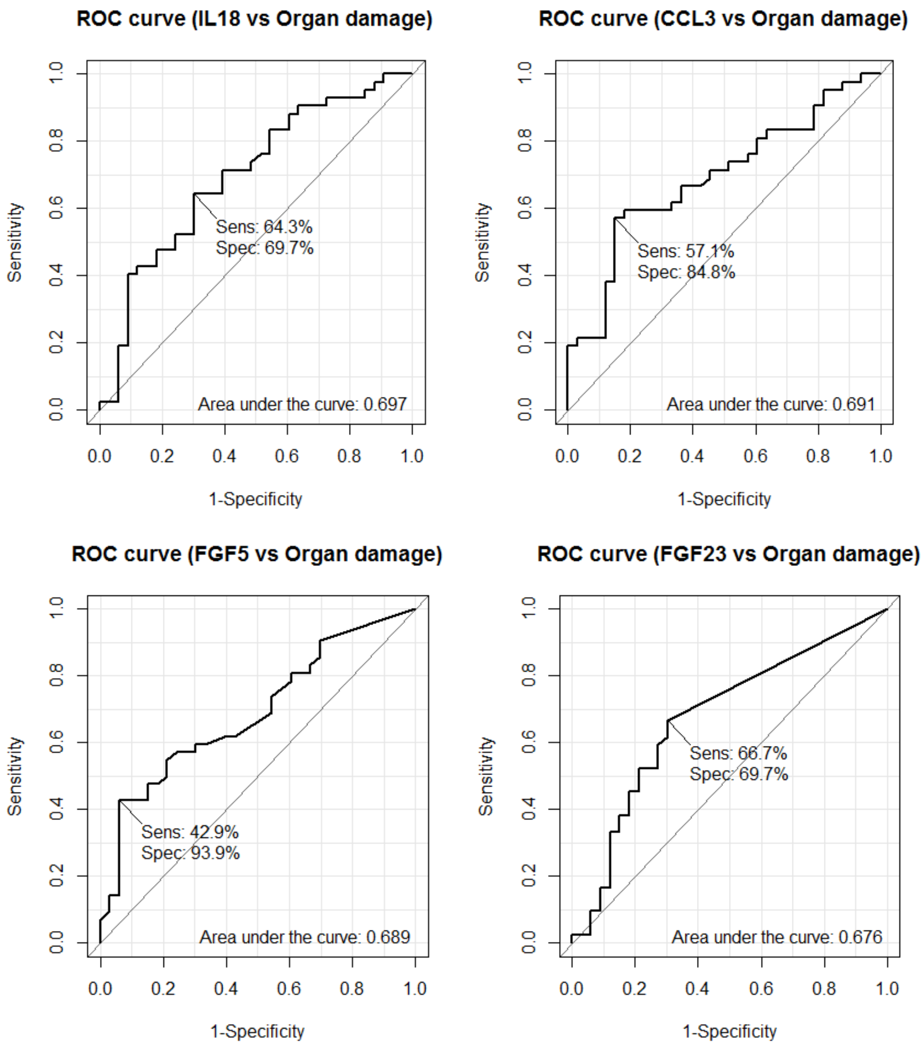
ROC curve (IL18 vs Organ damage)
Sens: 64.3%
Spec: 69.7%
Area under the curve: 0.697
Sensitivity
1-Specificity
ROC curve (CCL3 vs Organ damage)
Sens: 57.1%
Spec: 84.8%
Area under the curve: 0.691
Sensitivity
1-Specificity
ROC curve (FGF5 vs Organ damage)
Sens: 42.9%
Spec: 93.9%
Area under the curve: 0.689
Sensitivity
1-Specificity
ROC curve (FGF23 vs Organ damage)
Sens: 66.7%
Spec: 69.7%
Area under the curve: 0.676
Sensitivity
1-Specificity

b)

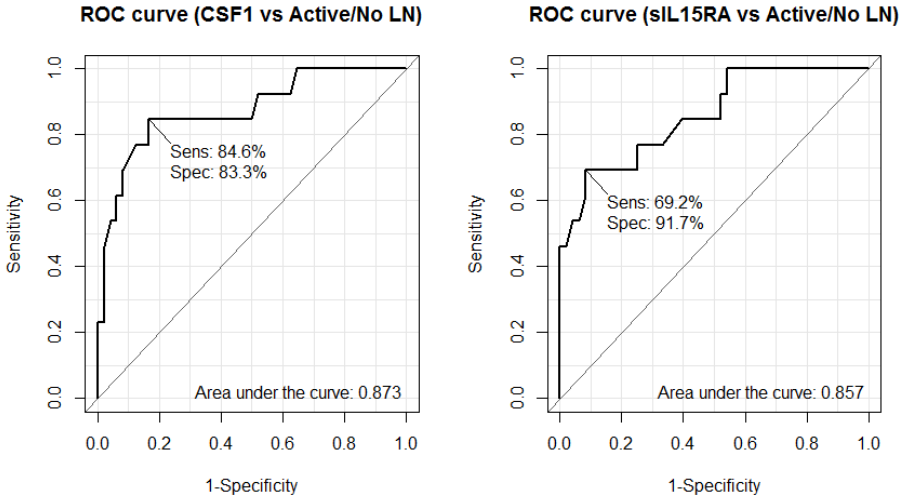
ROC curve (CSF1 vs Active/No LN)
Sens: 84.6%
Spec: 83.3%
Area under the curve: 0.873
Sensitivity
1-Specificity
ROC curve (sIL15RA vs Active/No LN)
Sens: 69.2%
Spec: 91.7%
Area under the curve: 0.857
Sensitivity
1-Specificity

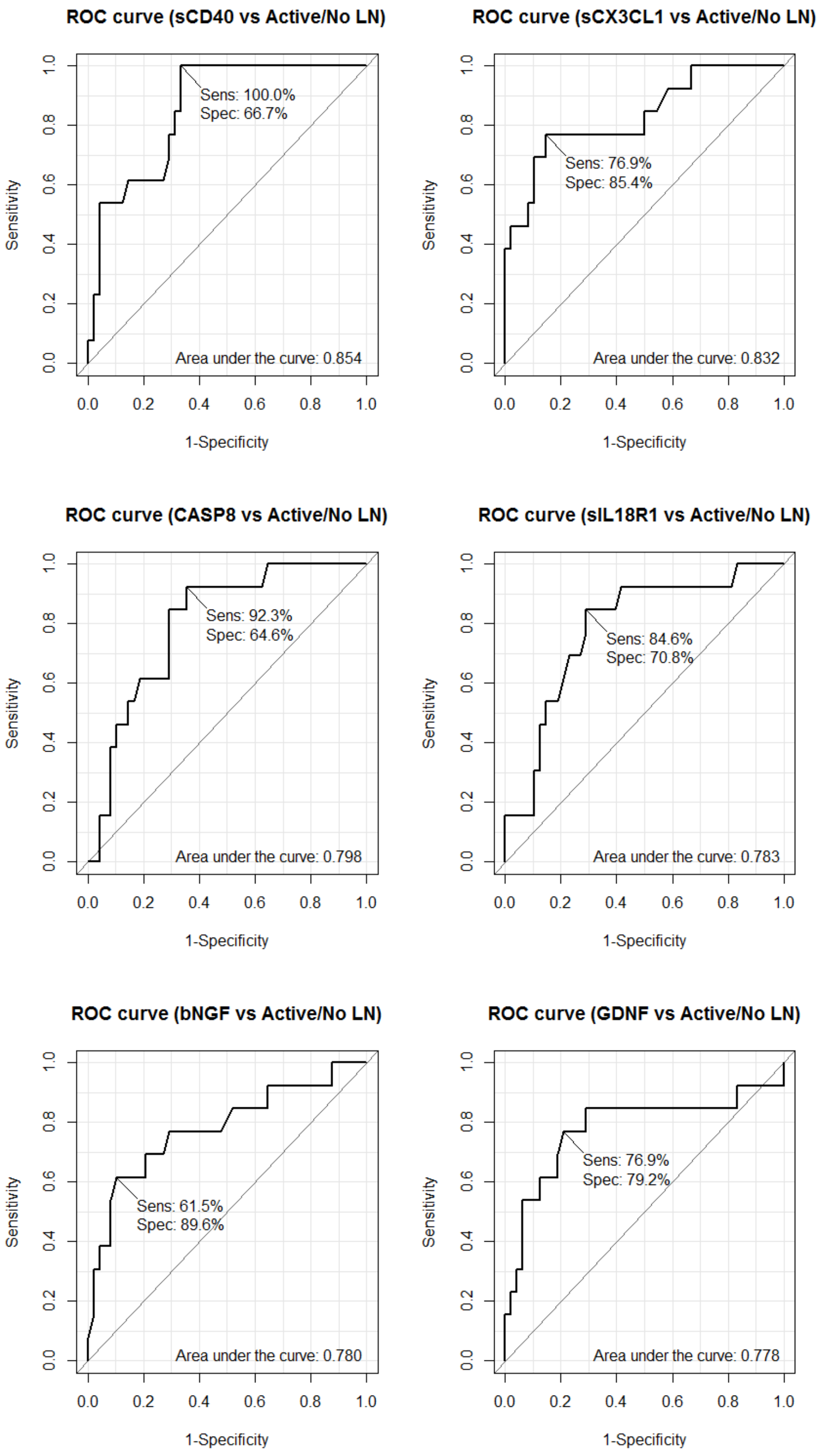
ROC curve (sCD40 vs Active/No LN)
Sens: 100.0%
Spec: 66.7%
Area under the curve: 0.854
ROC curve (sCX3CL1 vs Active/No LN)
Sens: 76.9%
Spec: 85.4%
Area under the curve: 0.832
ROC curve (CASP8 vs Active/No LN)
Sens: 92.3%
Spec: 64.6%
Area under the curve: 0.798
ROC curve (sIL18R1 vs Active/No LN)
Sens: 84.6%
Spec: 70.8%
Area under the curve: 0.783
ROC curve (bNGF vs Active/No LN)
Sens: 61.5%
Spec: 89.6%
Area under the curve: 0.780
ROC curve (GDNF vs Active/No LN)
Sens: 76.9%
Spec: 79.2%
Area under the curve: 0.778
Sensitivity
1-Specificity
0.0
0.2
0.4
0.6
0.8
1.0

c)

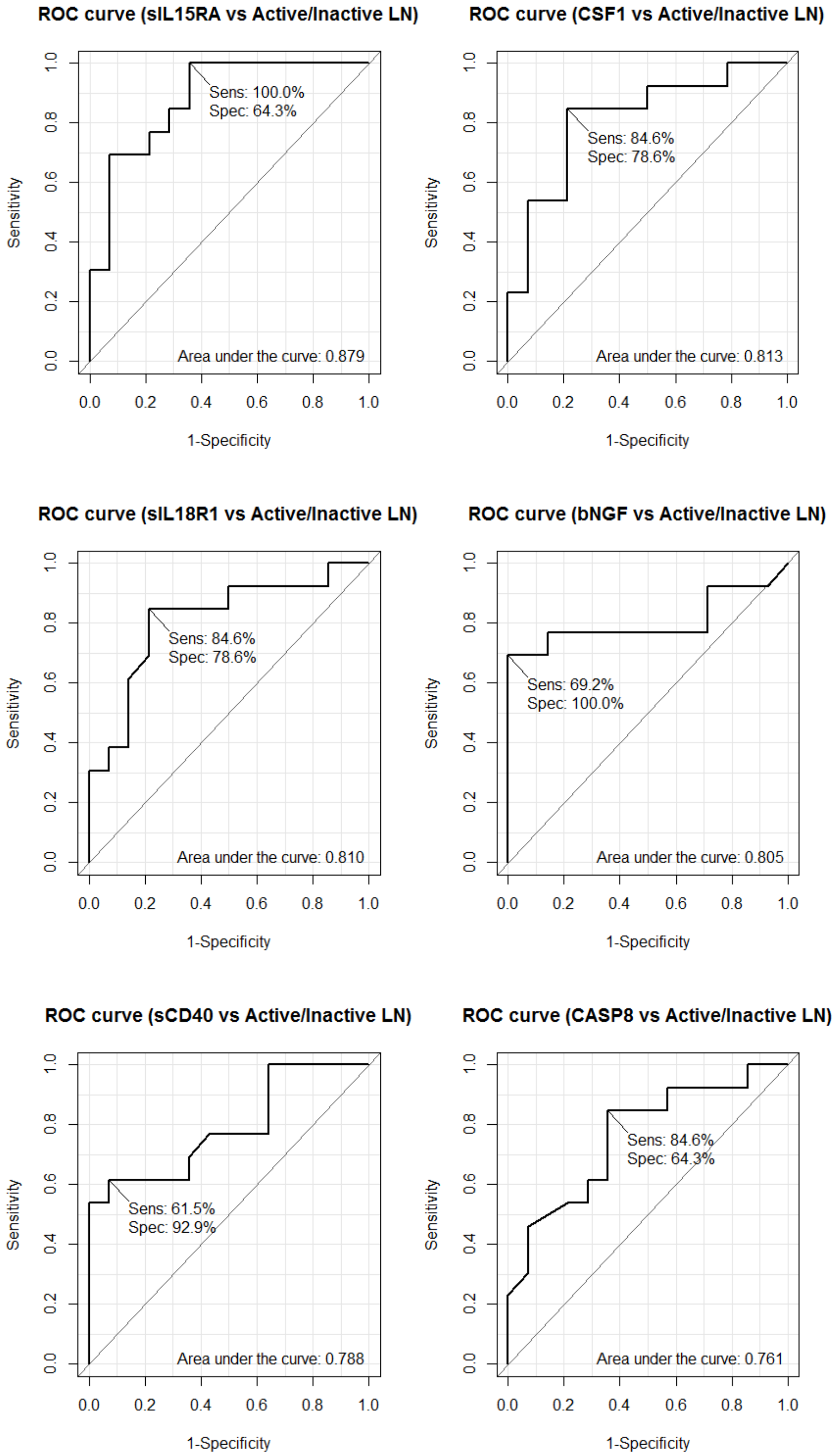
ROC curve (sIL15RA vs Active/Inactive LN)
Sens: 100.0%
Spec: 64.3%
Area under the curve: 0.879
Sensitivity
1-Specificity
ROC curve (CSF1 vs Active/Inactive LN)
Sens: 84.6%
Spec: 78.6%
Area under the curve: 0.813
Sensitivity
1-Specificity
ROC curve (sIL18R1 vs Active/Inactive LN)
Sens: 84.6%
Spec: 78.6%
Area under the curve: 0.810
Sensitivity
1-Specificity
ROC curve (bNGF vs Active/Inactive LN)
Sens: 69.2%
Spec: 100.0%
Area under the curve: 0.805
Sensitivity
1-Specificity
ROC curve (sCD40 vs Active/Inactive LN)
Sens: 61.5%
Spec: 92.9%
Area under the curve: 0.788
Sensitivity
1-Specificity
ROC curve (CASP8 vs Active/Inactive LN)
Sens: 84.6%
Spec: 64.3%
Area under the curve: 0.761
Sensitivity
1-Specificity

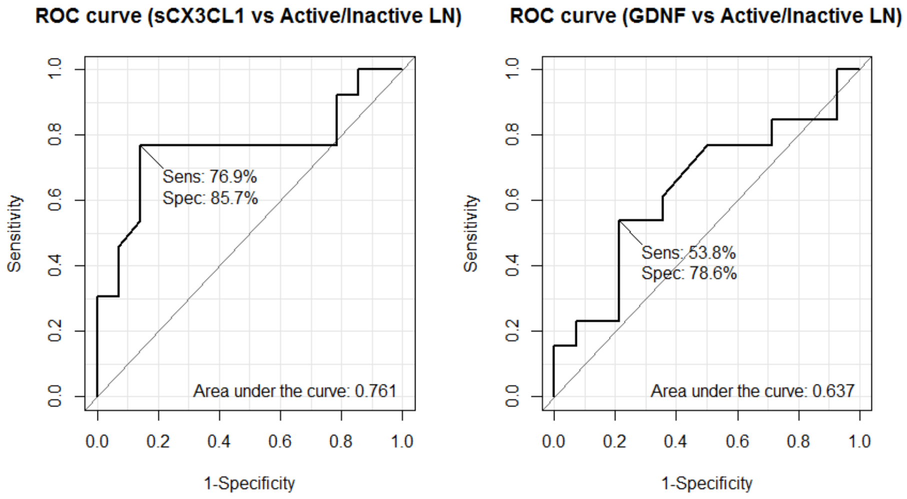

ROC curve (sCX3CL1 vs Active/Inactive LN)
Sensitivity
1-Specificity
Sens: 76.9%
Spec: 85.7%
Area under the curve: 0.761
ROC curve (GDNF vs Active/Inactive LN)
Sensitivity
1-Specificity
Sens: 53.8%
Spec: 78.6%
Area under the curve: 0.637

Table S5 Parameters of ROC analysis of serum proteins for a) organ damage *vs* no organ damage, b) active LN *vs* no LN, and c) active LN *vs* inactive LN as assessed by the renal SLEDAI.
Values in parentheses are 95 % confidence intervals.

a) Organ damage

Protein	*Sensitivity (%)*	*Specificity (%)*	*AUC*	*Accuracy (%)*	*Positive predictive value (%)*	*Negative predictive value (%)*	*Positive likelihood ratio*	*Negative likelihood ratio*
**IL8**	71.4 (55.4-84.3)	81.8 (64.5-93.0)	0.784 (0.672-0.784)	76.0 (64.7-85.1)	83.3 (67.2-93.6)	69.2 (52.4-83.0)	3.9 (1.9-8.3)	0.35 (0.21-0.58)
**CCL2**	88.1 (74.4-96.0)	60.6 (42.1-77.1)	0.738 (0.618-0.738)	76.0 (64.7-85.1)	74.0 (59.7-85.4)	80.0 (59.3-93.2)	2.2 (1.4-3.5)	0.20 (0.08-0.47)
**IL6**	88.1 (74.4-96.0)	51.5 (33.5-69.2)	0.731 (0.614-0.731)	72.0 (60.4-81.8)	69.8 (55.7-81.7)	77.3 (54.6-92.2)	1.8 (1.3-2.6)	0.23 (0.10-0.56)
**CCL11**	83.3 (68.6-93.0)	57.6 (39.2-74.5)	0.727 (0.612-0.727)	72.0 (60.4-81.8)	71.4 (56.7-83.4)	73.1 (52.2-88.4)	2.0 (1.3-3.0)	0.29 (0.14-0.60)
**FGF21**	54.8 (38.7-70.2)	87.9 (71.8-96.6)	0.723 (0.606-0.723)	69.3 (57.6-79.5)	85.2 (66.3-95.8)	60.4 (45.3-74.2)	4.5 (1.7-11.8)	0.51 (0.36-0.73)
**MMP10**	59.5 (43.3-74.4)	75.8 (57.7-88.9)	0.706 (0.588-0.706)	66.7 (54.8-77.1)	75.8 (57.7-88.9)	59.5 (43.3-74.4)	2.5 (1.3-4.7)	0.53 (0.35-0.81)
**IL18**	64.3 (48.0-78.4)	69.7 (51.3-84.4)	0.697 (0.575-0.697)	66.7 (54.8-77.1)	73.0 (55.9-86.2)	60.5 (43.4-76.0)	2.1 (1.2-3.7)	0.51 (0.32-0.82)
**CCL3**	57.1 (41.0-72.3)	84.8 (68.1-94.9)	0.691 (0.570-0.691)	69.3 (57.6-79.5)	82.8 (64.2-94.2)	60.9 (45.4-74.9)	3.8 (1.6-8.8)	0.51 (0.35-0.74)
**FGF5**	42.9 (27.7-59.0)	93.9 (79.8-99.3)	0.689 (0.569-0.689)	65.3 (53.5-76.0)	90.0 (68.3-98.8)	56.4 (42.3-69.7)	7.1 (1.8-28.3)	0.61 (0.46-0.80)
**FGF23**	66.7 (50.5-80.4)	69.7 (51.3-84.4)	0.676 (0.558-0.676)	68.0 (56.2-78.3)	73.7 (56.9-86.6)	62.2 (44.8-77.5)	2.2 (1.3-3.9)	0.48 (0.30-0.78)

b) Active LN *vs* no LN

Protein	*Sensitivity*	*Specificity*	*AUC*	*Accuracy (%)*	*Positive*	*Negative*	*Positive*	*Negative*

	(%)	*(%)*			*predictive value (%)*	*predictive value (%)*	*likelihood ratio*	*likelihood ratio*
**CSF1**	84.6 (54.6-98.1)	83.3 (69.8-92.5)	0.873 (0.753-0.873)	83.6 (71.9-91.8)	57.9 (33.5-79.7)	95.2 (83.8-99.4)	5.1 (2.6-10.0)	0.18 (0.05-0.66)
**sIL15RA**	69.2 (38.6-90.9)	91.7 (80.0-97.7)	0.857 (0.736-0.857)	86.9 (75.8-94.2)	69.2 (38.6-90.9)	91.7 (80.0-97.7)	8.3 (3.0-22.7)	0.34 (0.15-0.76)
**sCD40**	100 (75.3-100)	66.7 (51.6-79.6)	0.854 (0.754-0.854)	73.8 (60.9-84.2)	44.8 (26.4-64.3)	100 (89.1-100)	3.0 (2.0-4.5)	0.00 (NA)
**sCX3CL1**	76.9 (46.2-95.0)	85.4 (72.2-93.9)	0.832 (0.693-0.832)	83.6 (71.9-91.8)	58.8 (32.9-81.6)	93.2 (81.3-98.6)	5.3 (2.5-11.1)	0.27 (0.10-0.73)
**Caspase 8**	92.3 (64.0-99.8)	64.6 (49.5-77.8)	0.798 (0.677-0.798)	70.5 (57.4-81.5)	41.4 (23.5-61.1)	96.9 (83.8-99.9)	2.6 (1.7-3.9)	0.12 (0.02-0.79)
**sIL18R1**	84.6 (54.6-98.1)	70.8 (55.9-83.0)	0.783 (0.644-0.783)	73.8 (60.9-84.2)	44.0 (24.4-65.1)	94.4 (81.3-99.3)	2.9 (1.8-4.8)	0.22 (0.06-0.79)
**bNGF**	61.5 (31.6-86.1)	89.6 (77.3-96.5)	0.780 (0.617-0.780)	83.6 (71.9-91.8)	61.5 (31.6-86.1)	89.6 (77.3-96.5)	5.9 (2.3-15.0)	0.43 (0.21-0.86)
**GDNF**	76.9 (46.2-95.0)	79.2 (65.0-90.0)	0.778 (0.596-0.778)	78.7 (66.3-88.1)	50.0 (27.2-72.8)	92.7 (80.1-98.5)	3.7 (2.0-6.9)	0.29 (0.11-0.79)

c) Active LN *vs* inactive LN

Protein	*Sensitivity (%)*	*Specificity (%)*	*AUC*	*Accuracy (%)*	*Positive predictive value (%)*	*Negative predictive value (%)*	*Positive likelihood ratio*	*Negative likelihood ratio*
**sIL15RA**	100 (75.3-100)	64.3 (35.1-87.2)	0.879 (0.750-0.879)	81.5 (61.9-93.7)	72.2 (46.5-90.3)	100 (66.4-100)	2.8 (1.4-5.7)	0 (NA)
**CSF1**	84.6 (54.6-98.1)	78.6 (49.2-95.3)	0.813 (0.643-0.813)	81.5 (61.9-93.7)	78.6 (49.2-95.3)	84.6 (54.6-98.1)	4.0 (1.4-11.1)	0.20 (0.05-0.72)
**sIL18R1**	84.6 (54.6-98.1)	78.6 (49.2-95.3)	0.810 (0.637-0.810)	81.5 (61.9-93.7)	78.6 (49.2-95.3)	84.6 (54.6-98.1)	4.0 (1.4-11.1)	0.20 (0.05-0.72)
**bNGF**	69.2 (38.6-90.9)	100 (76.8-100)	0.805 (0.609-0.805)	85.2 (66.3-95.8)	100 (66.4-100)	77.8 (52.4-93.6)	NA (NA)	0.31 (0.14-0.70)
**sCD40**	61.5	92.9	0.788	77.8	88.9	72.2	8.6	0.41

	(31.6-86.1)	(66.1-99.8)	(0.610-0.788)	(57.7-91.4)	(51.8-99.7)	(46.5-90.3)	(1.2-59.8)	(0.21-0.84)
**sCX3CL1**	76.9	85.7	0.761	81.5	83.3	80.0	5.4	0.27
	(46.2-95.0)	(57.2-98.2)	(0.559-0.761)	(61.9-93.7)	(51.6-97.9)	(51.9-95.7)	(1.4-20.1)	(0.10-0.74)
**Caspase 8**	84.6	64.3	0.761	74.1	68.8	81.8	2.4	0.24
	(54.6-98.1)	(35.1-87.2)	(0.575-0.761)	(53.7-88.9)	(41.3-89.0)	(48.2-97.7)	(1.1-5.0)	(0.06-0.91)
**GDNF**	53.8	78.6	0.637	66.7	70.0	64.7	2.5	0.59
	(25.1-80.8)	(49.2-95.3)	(0.416-0.637)	(46.0-83.5)	(34.8-93.3)	(38.3-85.8)	(0.82-7.7)	(0.31-1.1)

NA = not appropriate

Table S6 Parameters of ROC analysis of established markers for a) organ damage *vs* no organ damage, b) no LN *vs* active LN as assessed by the renal SLEDAI.

Values in parentheses are 95 % confidence intervals.

a) Organ damage

Marker	*Sensitivity (%)*	*Specificity (%)*	*AUC*	*Accuracy (%)*	*Positive predictive value (%)*	*Negative predictive value (%)*	*Positive likelihood ratio*	*Negative likelihood ratio*
**C3**	47.6	36.4	0.527	42.7	48.8	35.3	0.75	1.4
	(32.0-63.6)	(20.4-54.9)	(0.394-0.527)	(31.3-54.6)	(32.9-64.9)	(19.7-53.5)	(0.50-1.1)	(0.84-2.5)
**C4**	54.8	36.4	0.497	46.7	52.3	38.7	0.86	1.2
	(38.7-70.2)	(20.4-54.9)	(0.371-0.503)	(35.1-58.6)	(36.7-67.5)	(21.8-57.8)	(0.59-1.3)	(0.71-2.2)
**Anti dsDNA**	57.1	30.3	0.532	45.3	51.1	35.7	0.82	1.4
	(41.0-72.3)	(15.6-48.7)	(0.400-0.532)	(33.8-57.3)	(36.1-65.9)	(18.6-55.9)	(0.58-1.2)	(0.76-2.6)
**Proteinuria**	60.7	8.3	0.452	36.5	43.6	15.4	0.66	4.7
	(40.6-78.5)	(1.0-27.0)	(0.384-0.548)	(23.6-51.0)	(27.8-60.4)	(1.9-45.4)	(0.48-0.91)	(1.2-19.2
**ESR**	35.7	87.9	0.640	58.7	78.9	51.8	2.9	0.73
	(21.6-52.0)	(71.8-96.6)	(0.514-0.640)	(46.7-69.9)	(54.4-93.9)	(38.0-65.3)	(1.1-8.0)	(0.56-0.95)
**GF**	45.2	27.3	0.638	37.3	44.2	28.1	0.62	2.0
	(29.8-61.3)	(13.3-45.5)	(0.525-0.638)	(26.4-49.3)	(29.1-60.1)	(13.7-46.7)	(0.42-0.92)	(1.1-3.7)
	50.0	78.8	0.635	62.7	75.0	55.3	2.4	0.63

**Creatinine**	(34.2-65.8)	(61.1-91.0)	(0.509-0.635)	(50.7-73.6)	(55.1-89.3)	(40.1-69.8)	(1.1-4.9)	(0.48-0.90)
**Anti-nucleosome antibodies**	59.5 (43.3-74.4)	24.2 (11.1-42.3)	0.499 (0.365-0.499)	44.0 (32.5-55.9)	50.0 (35.5-64.5)	32.0 (14.9-53.5)	0.79 (0.57-1.1)	1.7 (0.82-3.4)
**ACLA IgG**	28.2 (15.0-44.9)	50.0 (30.6-69.3)	0.484 (0.368-0.516)	37.3 (25.8-50.0)	44.0 (24.4-65.1)	33.3 (19.6-49.5)	0.56 (0.30-1.1)	1.4 (0.94-2.2)
**ACLA IgM**	38.5 (23.4-55.4)	75.0 (55.1-89.3)	0.487 (0.370-0.513)	53.7 (41.1-66.0)	68.2 (45.1-86.1)	46.7 (31.7-62.1)	1.5 (0.72-3.3)	0.82 (0.59-1.1)
**B2GPI IgG**	24.0 (9.4-45.1)	44.4 (21.5-69.2)	0.399 (0.427-0.601)	32.6 (19.1-48.5)	37.5 (15.2-64.6)	29.6 (13.8-50.2)	0.43 (0.19-0.97)	1.7 (0.98-3.0)
**B2GPI IgM**	40.0 (21.1-61.3)	77.8 (52.4-93.6)	0.486 (0.335-0.514)	55.8 (39.9-70.9)	71.4 (41.9-91.6)	48.3 (29.4-67.5)	1.8 (0.67-4.8)	0.77 (0.51-1.2)

b) Active LN *vs* no LN

Marker	*Sensitivity (%)*	*Specificity (%)*	*AUC*	*Accuracy (%)*	*Positive predictive value (%)*	*Negative predictive value (%)*	*Positive likelihood ratio*	*Negative likelihood ratio*
**C3**	61.5 (31.6-86.1)	4.2 (0.51-14.3)	0.659 (0.467-0.659)	16.4 (8.2-28.1)	14.8 (6.6-27.1)	28.6 (3.7-71.0)	0.64 (0.42-0.99)	9.2 (2.01-42.2)
**C4**	46.2 (19.2-74.9)	20.8 (10.5-35.0)	0.599 (0.380-0.599)	26.2 (15.8-39.1)	13.6 (5.2-27.4)	58.8 (32.9-81.6)	0.58 (0.32-1.1)	2.6 (1.2-5.5)
**Anti dsDNA**	38.5 (13.9-68.4)	43.8 (29.5-58.8)	0.547 (0.255-0.453)	42.6 (30.0-55.9)	15.6 (5.2-32.8)	72.4 (52.8-87.3)	0.68 (0.33-1.4)	1.4 (0.82-2.4)
**Proteinuria**	92.3 (64.0-99.8)	88.9 (70.8-97.6)	0.869 (0.711-0.869)	90.0 (76.3-97.2)	80.0 (51.9-95.7)	96.0 (79.6-99.9)	8.3 (2.8-24.4)	0.08 (0.01-0.57)
**ESR**	100 (75.3-100)	35.4 (22.1-50.5)	0.632 (0.480-0.632)	49.2 (36.1-62.3)	29.5 (16.8-45.2)	100 (80.5-100)	1.6 (1.3-1.9)	0.0 (NA)
**GF**	38.5 (13.9-68.4)	10.4 (3.47-22.7)	0.721 (0.539-0.721)	16.4 (8.15-28.1)	10.4 (3.47-22.7)	38.6 (13.9-68.4)	0.43 (0.21-0.86)	5.9 (2.3-15.0)
**Creatinine**	69.2 (38.6-90.9)	93.8 (82.8-98.7)	0.697 (0.466-0.697)	88.5 (77.8-95.3)	75.0 (42.8-94.5)	91.8 (80.4-97.7)	11.1 (3.5-35.1)	0.33 (0.14-0.74)

**Anti-nucleosome antibodies**	61.5 (31.6-86.1)	68.8 (53.7-81.3)	0.632 (0.454-0.632)	67.2 (54.0-78.7)	34.8 (16.4-57.3)	86.8 (71.9-95.6)	2.0 (1.1-3.6)	0.56 (0.27-1.1)
**ACLA IgG**	76.9 (46.2-95.0)	35.7 (21.6-52.0)	0.494 (0.331-0.506)	45.5 (32.0-59.4)	27.0 (13.8-44.1)	83.3 (58.6-96.4)	1.2 (0.82-1.7)	0.65 (0.22-1.9)
**ACLA IgM**	61.5 (31.6-86.1)	61.9 (45.6-76.4)	0.451 (0.363-0.549)	61.8 (47.7-74.6)	33.3 (15.6-55.3)	83.9 (66.3-94.5)	1.6 (0.91-2.9)	0.62 (0.30-1.3)
**B2GPI IgG**	54.5 (23.4-83.3)	65.4 (44.3-82.8)	0.455 (0.362-0.545)	62.2 (44.8-77.5)	40.0 (16.3-67.7)	77.3 (54.6-92.2)	1.6 (0.74-3.4)	0.70 (0.34-1.4)
**B2GPI IgM**	36.4 (10.9-69.2)	80.8 (60.6-93.4)	0.491 (0.294-0.509)	67.6 (50.2-82.0)	44.4 (13.7-78.8)	75.0 (55.1-89.3)	1.9 (0.62-5.7)	0.79 (0.49-1.3)

NA = not appropriate

GF = Glomerular filtration

ESR = Erythrocyte sedimentation rate

References:

1 Cervera R, Doria A, Amoura Z, Khamashta M, Schneider M, Guillemin F, et al. Patterns of systemic lupus erythematosus expression in Europe. Autoimmun Rev. 2014;13:621-9.

2 Al Sawah S, Zhang X, Zhu B, Magder LS, Foster SA, Iikuni N, et al. Effect of corticosteroid use by dose on the risk of developing organ damage over time in systemic lupus erythematosus — the Hopkins Lupus Cohort. Lupus Sci Med. 2015;2:e000066.
